# Identification and evolution of the plant sulfotransferase family

**DOI:** 10.1186/s12864-025-12117-4

**Published:** 2025-10-08

**Authors:** Shuangxin Han, Zhujun Chen, Quanlong Liu, Yueying Ding, Jia Wang, Hongbo Liu, Junyang Zou, Zhiying Hong, Hongmei Zhang, Wenping Yang, Lan Zhang, Hongwei Liu, Min Yuan

**Affiliations:** 1 College of Life Sciences, North China University of Science and Technology, Tangshan, 063210 Hebei China; 2 Institute of Biology, Hebei Academy of Sciences, Shijiazhuang, 050011 Hebei China

**Keywords:** Sulfotransferases, Origin, Green plants, HGT, TD, Lineage-specific

## Abstract

Sulfotransferases (SOTs, EC 2.8.2.-), which catalyze sulfate conjugation reactions, are widespread across prokaryotes and eukaryotes. However, the origin, classification and evolution history of SOTs in plants are not as well understood as those in animals and bacteria. In this study, a systematic analysis of an array of sequenced genomes revealed that SOTs were ubiquitously distributed in green plants (Viridiplantae). Phylogenetic analysis classified plant SOTs into three subfamilies, including SULTs (soluble sulfotransferases), TPSTs (tyrosylprotein sulfotransferases), and NFSTs (nodulation factor sulfotransferase). Notably, CHSTs (carbohydrate sulfotransferases), abundant in animals, algae and bacteria, were not found in land plants. High-throughput screening algorithms, phylogenetic and gene structure analyses indicated that land plants might acquire NFSTs through horizontal gene transfer (HGT) from bacteria to green algae. In contrast to the low gene number of TPSTs and NFSTs in land plants, the number of SULTs varied greatly among species. The absence of SULTs resulted in a significantly reduced gene number of SOTs in Cucurbitaceae, whereas the recent expansion of SULTs, mainly driven by tandem duplication (TD), caused a significant increase in SOT gene number in Begoniaceae. The significant variation in the gene number of SULTs across species, along with their evolutionary branching patterns, indicated lineage-specific duplication or contraction of SULTs, which profoundly influenced the production of sulfated metabolites during the diversification of monocots and core eudicots. This study provided the first comprehensive phylogenetic, classification and evolutionary analysis of SOTs in green plants across a broad taxonomic range.

**Supplementary Information:**

The online version contains supplementary material available at 10.1186/s12864-025-12117-4.

## Introduction

Sulfur is essential for all organisms as a constituent element of multiple crucial molecules, such as the S-containing amino acids (cysteine and methionine), coenzymes and metal cofactors (coenzyme-A and iron-sulfur Fe-S), and secondary metabolites [1, 2]. It is also a constituent of sulfate esters in numerous cellular metabolites, proteins, and carbohydrates. In this case, sulfate ion is transferred to a variety of substrates in the so-called sulfation reaction. Since a negatively charged sulfuryl group (SO3^−^) conjugated to an acceptor substrate, sulfation generally increases the water solubility of the substrate, thereby altering the biological activity and regulating the physiological functions of the substrate molecules. Sulfotransferases (SOTs) facilitate the transfer of a hydrophilic sulfonic acid group (-SO_3_H) from the universal sulfate donor, 3’-phosphoadenosine-5’-phosphosulfate (PAPS), onto a hydroxyl or amino group of the acceptor substrates. Hence, most of SOTs are PAPS-dependent sulfotransferases [3–6].

SOTs have been found to exist in both eukaryotes and prokaryotes, across animals, plants, bacteria and some viruses. In mammals, SOTs are classified into three major groups, including SULTs (soluble sulfotransferases), CHSTs (carbohydrate sulfotransferases), and TPSTs (tyrosylprotein sulfotransferases), which have distinct substrate preferences and subcellular localizations. SULTs are cytosolic-located sulfotransferases and prefer for sulfating low-molecular-weight metabolites, such as endocrine hormones, polyphenols and drugs. CHSTs and TPSTs are membrane-bound sulfotransferases and prefer for sulfating glycosaminoglycans and proteins, respectively [5, 7–10]. In plants, sulfation appears to play an important role in plant growth, development and stress responses [11–13]. However, even in the model plant *Arabidopsis thaliana*, SOTs have not been fully characterized on the classification, function and substrate specificity.

The first functionally characterized SOT protein from *Arabidopsis thaliana* is AthSOT12 (AT2G03760). Its recombinant protein exhibited 11- and 12-hydroxyjasmonate sulfation in vitro, and the expression of AthSOT12 was induced by methyljasmonate and 12-hydroxyjasmonate treatment [14, 15]. Two SOTs (BNST3 and BNST4) from *Brassica napus* were enzymatically characterized to catalyze the sulfation of brassinosteroids with a preference for 24-epicathasterone in vitro. Since the sulfated brassinosteroids become biologically inactive, BNST3 and BNST4 were hypothesized to be involved in brassinosteroids inactivation [16]. Until now, a total of 22 SOT genes (AthSOT1-AthSOT22) were identified through genome-wide analysis based on the sequence or function similarities. AthSOT1-AthSOT18 contain the Sulfotransfer_1 domain (PF00685) and are speculated as cytosolic-located sulfotransferases. AthSOT5, AthSOT8, AthSOT12 and AthSOT13 have been characterized as flavonoid sulfotransferases in vitro [17, 18]. AthSOT12 exhibited a broadest substrate specificity with substrate preference for flavonoids, brassinosteroids and salicylic acid [19]. Glucosinolates (Gls) are a group of best-characterized sulfur- and nitrogen-containing defense compounds in Brassicaceae. The last step of the Gl core structure biosynthesis is catalyzed by SOTs. AthSOT16, AthSOT17, and AthSOT18 have been characterized as the sulfotransferases involved in sulfation of desulfo-glucosinolates [20–22]. Additionally, three SOTs (AthSOT19-21) were identified as sulfotransferase and annotated as “nodulation-related protein” in NCBI, which is similar as bateria nodH (Nodulation factor sulfotransferase). However, the substrate information of AthSOT19-21 has not been reported yet. Later, a tyrosylprotein SOT protein (AthTPST) in *Arabidopsis thaliana* was identified, which could sulfate peptide hormones PSK and PSY1. AthTPST shows very low sequence similarity either with other AthSOTs or animal TPSTs, suggesting that plants have evolved plant-specific TPSTs which are distinct from their animal counterparts. Moreover, AthTPST is found to be the only Arabidopsis SOT protein possessing a Sulfotrans-fer_2 domain (PF03567) instead of a Sulfotransfer_1 domain (PF00685) [23, 24].

Recently, genome-wide identification of the SOTs was performed in a limited number of species, including Arabidopsis (*Arabidopsis thaliana*), Chinese cabbage (*Brassica napus*), potato (*Solanum tuberosum*), rice (*Oryza sativa*), cotton (*Gossypium*) and wheat [25–29]. However, most of the current studies on plant SOTs have still focused on SULTs with low-molecular-weight metabolites as substrates. Whether CHSTs exist in plants has not been reported so far. Multiple proteins with representative domains under different IPR numbers were associated to SOTs by function in the InterPro database, and small differences in the protein sequences led to wide variations in substrate specificity and big changes in sub-grouping, which made the classification and nomenclature of plant SOTs is relatively difficult. Hence, the origin and evolution history of plant SOTs is still opaque, due to the lack of comprehensive understanding on classification, substrates, and catalytic mechanisms and functions in plants.

Here, a comprehensive identification of the SOT family was conducted across a broad taxonomic range of green plants to elucidate its evolutionary origin. The first systematic phylogenetic and evolutionary analyses revealed that plant SOTs were divided into three subfamilies, including SULSTs, TPSTs, and NFSTs. Land plants lost CHSTs but acquired NFSTs through a HGT event from bacteria to green algae. SULTs underwent lineage-specific and species-specific evolution, resulting in a corresponding change in sulfated products, particularly in monocots and core eudicots. This study provided new insights into the origin and evolutionary history of the SOT gene family in plants.

## Results

### Identification of sulfotransferase (SOT) genes in plants

**Fig. 1:**
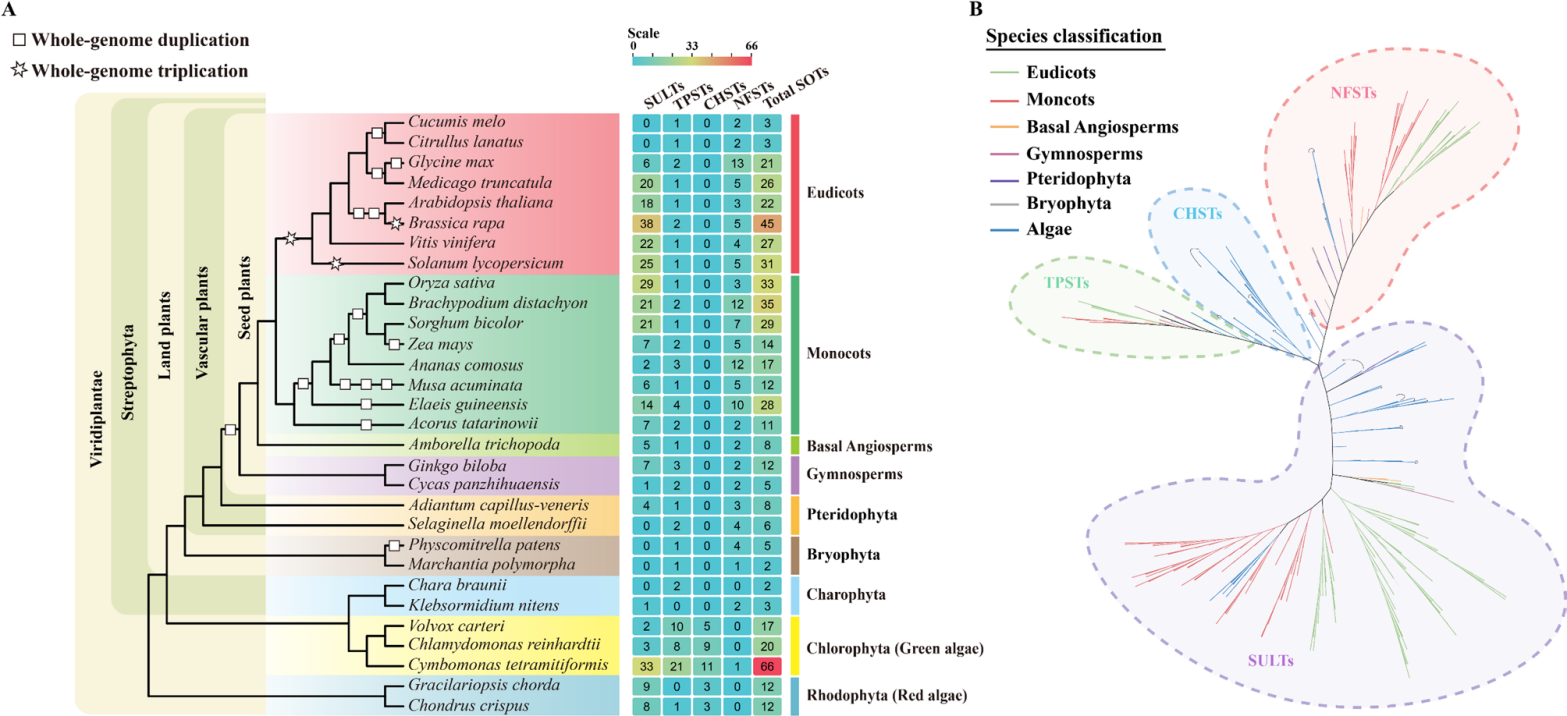
Genome-wide identification and phylogenetic investigation of the SOT gene family.**A** The taxonomic information and distribution of SOTs across plant kingdom were showed. The number of SOTs within each species was labeled and colored based on abundance. Subgroups are assigned based on the phylogenetic tree. **B** Unrooted phylogenetic tree of plant SOT genes. The tree was constructed using SOTs identified from 30 species in this study, including 2 species of red algae, 5 species of green algae, and 2 species of bryophyta, 2 species of pteridophyta, 2 species of gymnosperms, 1 species of basal angiosperms, 8 species of monocots, and 8 species of eudicots. The SOTs were divided into four major classes (SULTs, TPSTs, NFSTs and CHSTs), while CHSTs were only found in algae and absent in land plants. Colored branches were used to distinguish clades of eudicots, monocots, basal angiosperms, gymnosperms, pteridophyta, bryophyta, and algae. Some algal SOTs on the evolutionary tree that did not appear in the groups indicated by IPR numbers were indicated by parentheses

To determine the occurrence and distribution of SOTs in green plants, genome sequences of 30 species were analyzed, including 2 species of red algae, 5 species of green algae, and 23 species of land plants. A total of 534 SOTs were identified using combined Hidden Markov Model (HMM) and BLASTP searching methods. SOTs were widely distributed across green plants, including chlorophyta (green algae), bryophytes, ferns, gymnosperms, and angiosperms, as well as in rhodophyta (red algae). The results indicated an ancient origin of the SOT gene family. The number of SOTs greatly varied among different species. A high number of SOTs was detected in rhodophyta and chlorophyta. The number of SOTs decreased in charophyta, bryophytes, and ferns, and gradually increased in gymnosperms and basal angiosperms. The highest abundance of SOTs was found in monocots and core eudicots, such as in Brassicaceae, Poaceae, Fabaceae, and Solanaceae. However, a strikingly fewer number of SOTs was found in the *Cucumis melo* and *Citrullus lanatus* from Cucurbitaceae, with only 3 SOTs found in these two species, respectively (Fig. 1A, Additional file 1: Table S1).

### Phylogenetic analysis of sulfotransferase (SOT) genes in plants

To clarify the classification of plant SOTs, both unrooted and rooted phylogenetic trees were constructed using SOTs longer than 200 amino acids to ensure the accuracy of phylogenetic analysis (Fig. 1B, Additional file 2: Fig. S1). The subfamily information of SOTs in *A. thaliana* was analyzed and used as a reference. In *A. thaliana*, all the originally identified SOTs (AthSOT1-18) are cytosolic sulfotransferases (SULTs), which are believed to sulfating low-molecular metabolites. Another three SOTs (AthSOT19-21) were annotated as “Nodulation factor sulfotransferase (IPR052796)” in the InterPro database. Their prefer substrates are yet unknown. A new member (AthTPST), which can sulfate tyrosylproteins, was added into the SOT family by functional similarity not by sequence. AthTPST showed no sequence similarity either to SULTs or to human TPSTs, and was annotated as “Heparan sulphate 6-sulfotransferase/Protein-tyrosine sulfotransferase (IPR010635)” in the InterPro database. Based on the distribution of SOTs in major lineages of land plants and the classification of SOTs in *A. thaliana*, four major divisions were established, including SULTs, TPSTs, NFSTs, and an algae-specific subfamily. These algae-specific SOTs were designated as CHSTs (carbohydrate sulfotransferases) according to the nomenclature of SOTs sharing the same IPR numbers in metazoans. Although some algal SOTs with specific IPR numbers were grouped into the SULT subfamily based on the topology of the unrooted phylogenetic tree, they exhibited substantial divergence from plant SULTs, indicating the presence of special SULTs in algae, and an independent origin and evolutionary trajectories for algal and land plant SULTs, respectively (Fig. 1B, Additional file 2: Fig. S1, Additional file 1: Table S1). If algal SOTs were excluded, plant SOTs would be tightly clustered into three subfamilies, including SULTs, TPSTs, and NFSTs, suggesting that, unlike the diverse SOTs in algae, plant SOTs have undergone lineage-specific evolution. Furthermore, the topology of the evolutionary tree revealed a more recent origin of SULTs relative to TPSTs and NFSTs (Additional file 2: Fig. S2).

Additionally, the distribution of SOTs across subfamilies was determined and calculated for each species. Each subfamily exhibited distinct characteristics in terms of gene number, occurrence, and evolutionary branching. SULTs were widely distributed in red and green algae with diverse IPR numbers. In land plants, SULTs were absent in liverworts, mosses, scarce in gymnosperms, and became more abundant in angiosperms, especially in monocots and core eudicots, such as *Oryza sativa*, *Sorghum bicolor*, *Solanum lycopersicum*, *Brassica rapa* and so on (Fig. 1A). Surprisingly, an absence of SULTs was observed in *C. melo* and *C. lanatus* from Cucurbitaceae. In addition, SULTs within the same species were often closely clustered in the phylogenetic tree (Additional file 2: Fig. S3). The large variation in the gene number within species and the evolutionary branching characteristics indicated a lineage-specific duplication or contraction of SULTs during the diversification in monocots and core eudicots. TPSTs were maintained at low numbers across plant lineages, although slightly higher numbers were observed in algae, indicating the high conservation and no obvious expansion during its evolution. NFSTs are ubiquitously present at a low number across the plant kingdom but absent in rhodophyta (Fig. 1A).

Nodulation factors are key signaling molecules in symbiotic relationships between nitrogen-fixing bacteria and certain plants. Since the N-terminal sequences of NFSTs appeared to be present in bacterial NodH (lipo-oligosaccharide 6-O-sulfotransferase), which is involved in sulfating of nodulation factors, we proposed a possible evolutionary link between plant NFSTs and bacterial nodulation factor sulfotransferases. The occurrence of NFSTs was investigated on a large scale by querying the InterPro database using the corresponding IPR number (IPR052796). A dataset of 2,490 NFSTs were retrieved, including 453 from bacteria, 2,002 from viridiplantae, and the rest from archaea. Surprisingly, NFSTs were not identified from metazoans (Additional file 1: Tables S2-S3).

**Fig. 2:**
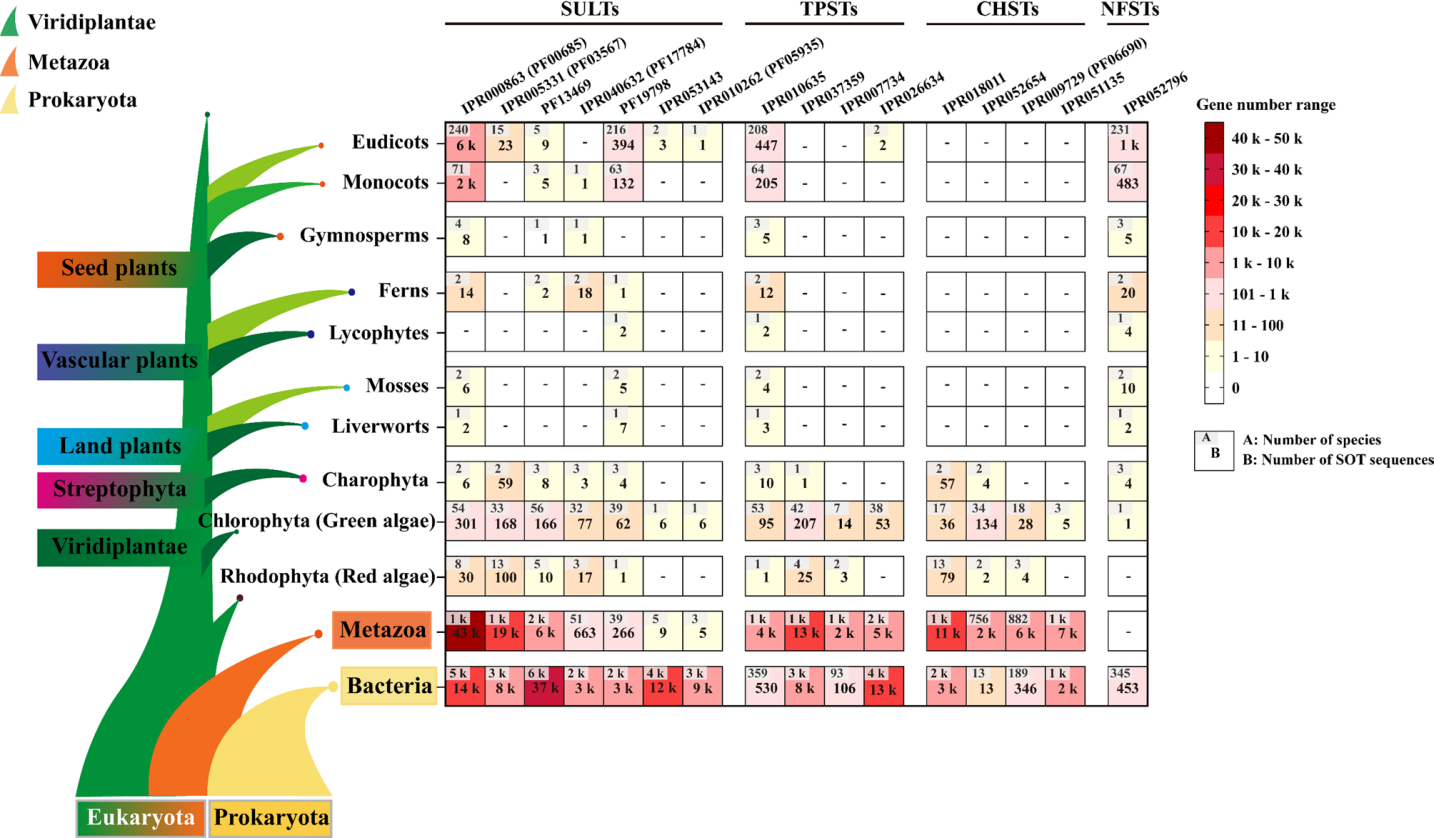
Distribution of SOT genes across viridiplantae, metazoa and bacteria genomes. All IPR numbers associated with SOTs were searched against the InterPro database (accessed and retrieved from December 28, 2024, to January 4, 2025) to identify the presence of SOTs in prokaryotes and eukaryotes. The number of SOTs and the number of the species possessing SOTs were presented within major clades and lineages of organisms. Numbers were colored based on abundance, and “-” denoted not detected. The plant phylogeny was drawn according to a previous study (Yang et al., 2023)

In addition, the occurrence of other subfamilies of SOTs were also investigated on a large scale by querying the InterPro database using corresponding IPR numbers. Besides NFSTs, multiple IPR numbers corresponded to the same SOT subfamily. CHSTs were widely distributed in metazoa, bacteria, rhodophyta, and chlorophyta, whereas entirely absent in streptophyta, including charophyta. SULTs under IPR000863 and TPSTs under IPR 010635 possessed the most ubiquitous occurrence, and have been found in viridiplantae, metazoa, algae, and bacteria. In contrast, SULTs and TPSTs under other IPR numbers were barely found in land plants (Fig. 2, Additional file 1: Tables S4-18).

Taken together, we found that the distribution patterns of CHSTs and NFSTs were significantly different, with CHSTs entirely absent in land plants and NFSTs entirely absent in animals, respectively. By contrast, algae and bacteria possessed all the four subfamilies of SOTs, including SULTs, TPSTs, CHSTs, and NFSTs.

### The NFST subfamily genes of plants might originate from bacteria through horizontal gene transfer

**Fig. 3:**
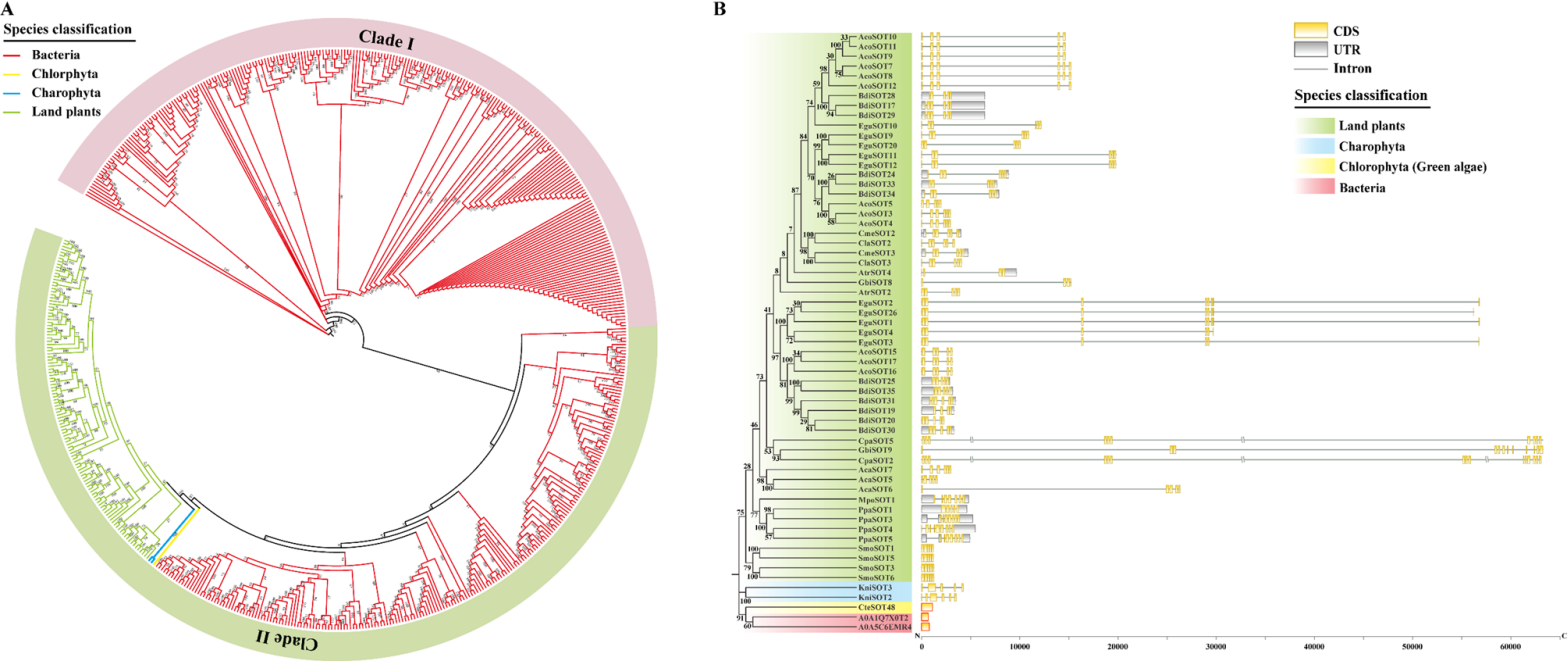
Plant SOTs were transferred from bacteria by a horizontal gene transfer (HGT) event.**A** Phylogenetic tree within the NFST subfamily. NFSTs from 30 representative plants identified in this study, plus bacteria NFSTs obtained by searching against the InterPro database under IPR052796, were collected and constructed the phylogenetic tree. Colored branches were used to distinguish clades of land plants, charophyta, chlorphyta, and bacteria. The phylogenetic tree was clearly separated into two clades (Clade I and II). **B** Gene structure analysis of NFSTs. Gene structure analysis of NFSTs from charophyta, chlorphyta, bacteria, and some representative land plants. Yellow and gray boxes indicated the coding sequences (CDS) and untranslated regions (UTR), respectively. The lines between boxes represented introns

Since NFSTs are prevalent in green plants and bacteria, but entirely absent in metazoans, we wonder if the NFSTs in viridiplantae are outcomes of HGT (horizontal gene transfer) events from bacteria. To explore the evolutionary relationships between plant and bacterial NFSTs, a phylogenetic tree was constructed. Given that the evolutionary relationships of NFSTs in land plants are relatively well characterized, only NFSTs from selected representative plant species were included. Finally, a dataset comprising 100 NFSTs from rhodophyta, chlorophyta, and land plants, along with 453 bacterial NFSTs, was created for the phylogenetic tree construction. All the NFSTs in the phylogenetic tree was clearly separated into two clades (Clade I and II). Clade I consisted of bacterial NFSTs, whereas Clade II consisted of a mix of bacterial and plant SOTs supported by a high bootstrap value 90. Within Clade II, all plant NFSTs, including streptophyta and green algae, formed a distinct branch with strong bootstrap support value 96, and were further grouped with bacterial NFSTs, suggesting that plant NFSTs may have originated via horizontal gene transfer (HGT) from bacteria (Fig. 3A, Additional file 1: Table S3).

Furthermore, gene structure analysis revealed that introns were absent in bacterial and green algal *NFSTs*, but present in streptophyta *NFSTs*. The similar gene structures of bacterial and green algal *NFSTs* provided additional evidence for the transfer of NFSTs from bacteria to plant, suggesting that HGT of NFSTs likely occurred before plants invaded the land (green algae) (Fig. 3B).

High-throughput screening algorithms was employed to identify putative HGT genes of bacterial origin in *Cymbomonas tetramitiformis*. The results showed that 1,973 putative HGT genes of bacterial origin in *C. tetramitiformis* were identified, with 1,514 and 459 putative HGT genes distributed in the high-confidence and medium-confidence categories, respectively. CteSOT48 (NFST family member in *C. tetramitiformis*) was detected in the medium-confidence category (Additional file 2: Fig. S4, Additional file 1: Table S19-20). Therefore, we examined the blast result of CteSOT48, and found that CteSOT48 displayed specific alignment only with high-confidence bacterial sequences in distal groups, with no detectable homology to any close-group organisms (Additional file 1: Table S21). These findings indicated that CteSOT48 might be acquired through horizontal gene transfer from bacteria. To confirm the bacterial origin of the NFST gene in *C. tetramitiformis*, further experimental validation should be performed through its introduction into the donor bacterial species, assessing both ancestral function retention and potential novel functions in the recipient host. However, the precise bacterial donor requires prior determination, as phylogenetic analysis showed plant NFSTs clustering with diverse bacterial species across different genera, and high-throughput screening revealed multiple candidate donors.

Since both the substrates of CHSTs in animals and NFSTs in bacteria are associated with glucose backbones, whether gaining NFSTs via HGT enabled the loss of CHSTs function in plants required to be further investigated through in vivo functional and catalytic mechanisms analyses.

### The SULT subfamily genes experienced lineage-specific evolution

Another interesting phenomenon caught our attention was that much fewer SOTs were identified in *C. melo* and *C. lanatus* from Cucurbitaceae. To confirm this result, SOTs were searched in 14 Cucurbitales species using combined Hidden Markov Model (HMM) and BLASTP searching methods, including 10 Cucurbitaceae species and 4 Begoniaceae species. Arabidopsis and grapes were used as outgroups. A total of 122 SOTs were identified in the 14 Cucurbitales species. A large variation in the number of SOTs was observed among the species from Cucurbitaceae and Begoniaceae under Cucurbitales. A strikingly fewer number of SOTs were found in all the 10 Cucurbitaceae species (3 or 6), while the SOTs were abundant in the Begoniaceae species (Additional file 1: Table S22).

**Fig. 4:**
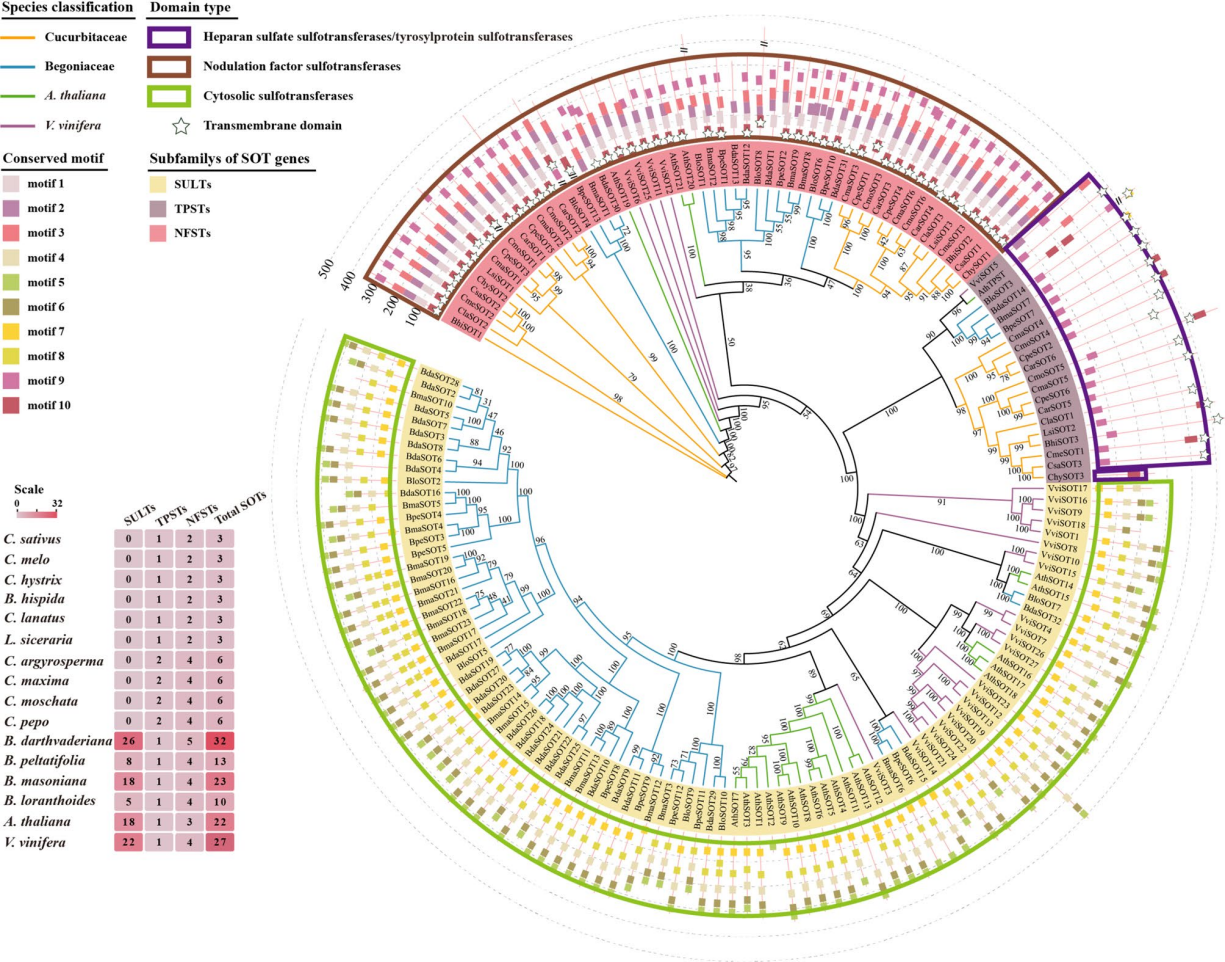
Phylogeny and conserved domains of SOTs from Cucurbitales. Phylogenetic tree of SOTs from 14 species in Cucurbitales were built. The SOTs sequences from Arabidopsis and grape were used as outgroups. Colored branches were used to distinguish clades of Cucurbitaceae, Begoniaceae, Arabidopsis and grape. The protein names in the tree were indicated by colored symbols corresponding to three subfamilies, including SULTs (yellow), TPSTs (brown), and NFSTs (red). Conserved motifs and domains of SOTs in different subfamilies were shown on the outer panel of the evolutionary tree. Ten color solid boxes indicated different kinds of motifs. The positions of conserved domains were indicated by brown, purple and green rectangular frames, which correspond to “Nodulation factor sulfotransferase”, “Heparan sulphate 6-sulfotransferase/Protein-tyrosine sulfotransferase”, and “cytosolic sulfotransferases” for NFSTs, TPSTs, and SULTs, respectively. Hollow five-pointed stars indicated the transmembrane domains in the NFST and TPST subfamilies. The number of SOT genes distributed in each of classes was calculated of each species, and shown using a heat map in the lower left corner

To explore the reasons for the significant decrease of SOT members in Cucurbitaceae, a phylogenetic tree was constructed using the SOTs identified from Cucurbitales. The SOTs from Cucurbitales were exactly grouped into three subfamilies that correspond to the SULT, TPST and NFST subfamilies. The distribution of SOTs across subfamilies was determined and calculated for each species in both Cucurbitaceae and Begoniaceae. SOTs from the TPST and NFST subfamilies were detected at a low number (usually less than 5) in both Cucurbitaceae and Begoniaceae, indicating high conservation and no obvious expansion. However, SOTs from the SULT subfamily showed divergent evolutionary trajectories in Cucurbitaceae and Begoniaceae under the same Cucurbitales. In Begoniaceae, the number of SULTs was significantly higher than TPSTs and NFSTs. The SULT subfamily radiated out from ancestral TPST and NFST subfamilies, with SULTs from the same species clustering together in the phylogenetic tree, suggesting lineage-specific and species-specific evolution of the SULT subfamily across different plant species. Surprisingly, the SULT subfamily genes were entirely absent in the 10 Cucurbitaceae species, which resulted in the low number of total SOTs (Fig. 4, Additional file 1: Table S22).

To further characterize SOT proteins in Cucurbitaceae and Begoniaceae, conserved domains and motifs were analyzed by querying InterPro database and using the MEME software, respectively. Group-specific domain types were clearly observed across the three subfamilies, further validating the phylogenetic tree topology. Three critical SOT-related domains, Sulfotransfer_1 (IPR000863, PF00685), Heparan_SO4-6-sulfoTrfase (IPR010635) and Nod_factor_sulfotransferase (IPR052796) were identified in Cucurbitaceae and Begoniaceae SOTs, corresponding to the SULT, TPST, and NFST subfamilies, respectively. Consistently, the motif patterns across the three subfamilies were also group-specific. Motifs 4, 5, 6, 7, and 8 corresponded to Sulfotransfer_1 (IPR000863, PF00685). Motifs 1, 2, 3, 9 and 10 corresponded to Nod_factor_sulfotransferase (IPR010635). Heparan_SO4-6-sulfoTrfase (IPR052796) domain harbored the fewest motifs, including motifs 9 and 10. Subcellular localization analysis using THMHH revealed that transmembrane domains were detected in TPSTs and NFSTs but not in SULTs. SOTs from all the 10 Cucurbitaceae species were membrane-associated sulfotransferases, providing additional evidence for the absence of cytosolic SULTs in these species (Fig. 4).

**Fig. 5:**
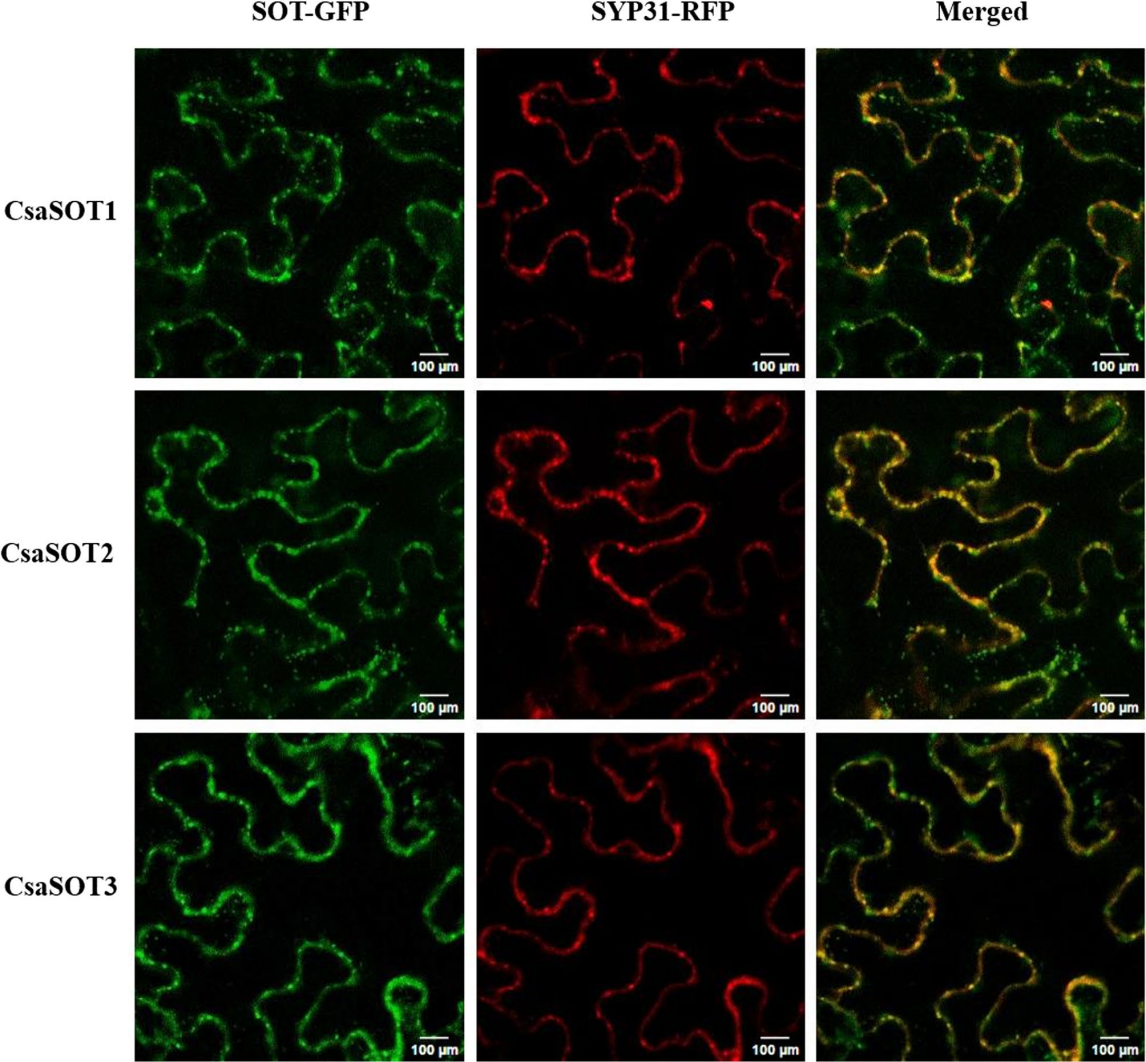
Subcellular localizations of the CsaSOT proteins. Subcellular localization patterns of CsaSOT-GFP fusion proteins were checked under a laser scanning confocal microscope. SYP31-mRFP was used as a known *cis*-Golgi marker. Bars, 100 μm. CsaSOTs were localized in the Golgi apparatus as beaded filaments

GFP-fused cucumber SOTs were transiently expressed in tobacco leaves to verify their subcellular localization. It has been previously noted that membrane-associated sulfotransferases were localized in *cis*-Golgi. Our results showed that CsaSOTs were localized in the Golgi apparatus as beaded filaments, most likely in *cis*-Golgi (Fig. 5). The results are largely consistent with the topology of the phylogenetic tree.

### Gene gain and loss analysis of the SOT gene family

**Fig. 6:**
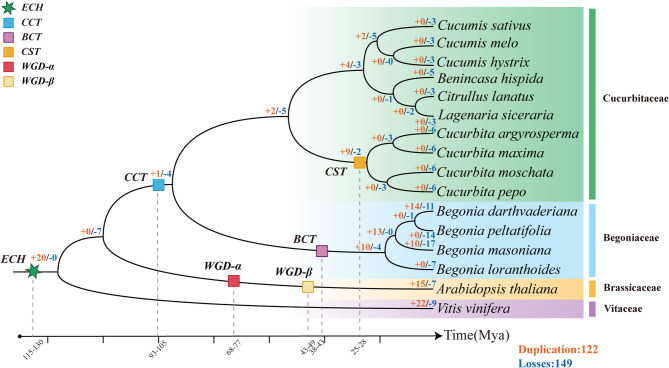
Duplication and loss analyses of SOTs in Cucurbitales. Duplication and loss analyses of SOTs in Cucurbitales. The “+” and “–” symbols with values denoted duplication and loss of the SOT genes, respectively. Square and six-pointed stars of different colors in the species-tree indicated the genome-wide tetraploidy or hexaploidy events, with occurrence time at the bottom timeline. CCT: Cucurbitaceae common tetraploidization, BCT: Begoniaceae common tetraploidization, CST: Cucurbita-specific tetraploidization, ECH: core-eudicot-common hexaploidization

To explore the reasons for the lack of SULTs in Cucurbitaceae, duplication and loss events of the SOTs were examined during the evolution of all 14 Cucurbitales species. Arabidopsis and grapes were used as reference. Given the absence of SULTs in Cucurbitaceae and their presence in Begoniaceae, we speculated that SULTs experienced differentiation in Cucurbitales. Consistently, gene loss events occurred more frequently than gene replication events in Cucurbitaceae. No gene duplications were detected during the recent species differentiation and formation of the 10 Cucurbitaceae species, whereas an average of 3–6 SOT genes were lost in each species during this step. For example, after the Cucurbita-specific polyploidizations event (CST), 6 SOT genes were lost in each of the species among *Cucurbita. argyrosperma*, *Cucurbita. moschata*, *Cucurbita. maxima* and *Cucurbita. pepo*. In contrast, both gene duplications and loss were observed in Begoniaceae during the same period (Fig. 6).

#### Synteny analysis of SOT genes

Since SULTs were absent in Cucurbitaceae but present in the closely related Begoniaceae, synteny analyses were performed between the Cucurbitaceae species (*C. sativus* and *C. maxima*) and grape (*V. vinifera*) to investigate the evolutionary history of the SOT gene family in Cucurbitaceae. Representative Begoniaceae species (*B. loranthoides* and *B. darthvaderiana*) were selected as the control due to the presence of SULTs in Begoniaceae. The genome of grape has not undergone recent whole-genome duplication events after the core-eudicot common hexaploidization event (ECH), and greatly preserved the chromosome structure of the most recent ancestral core-eudicots.

**Fig. 7:**
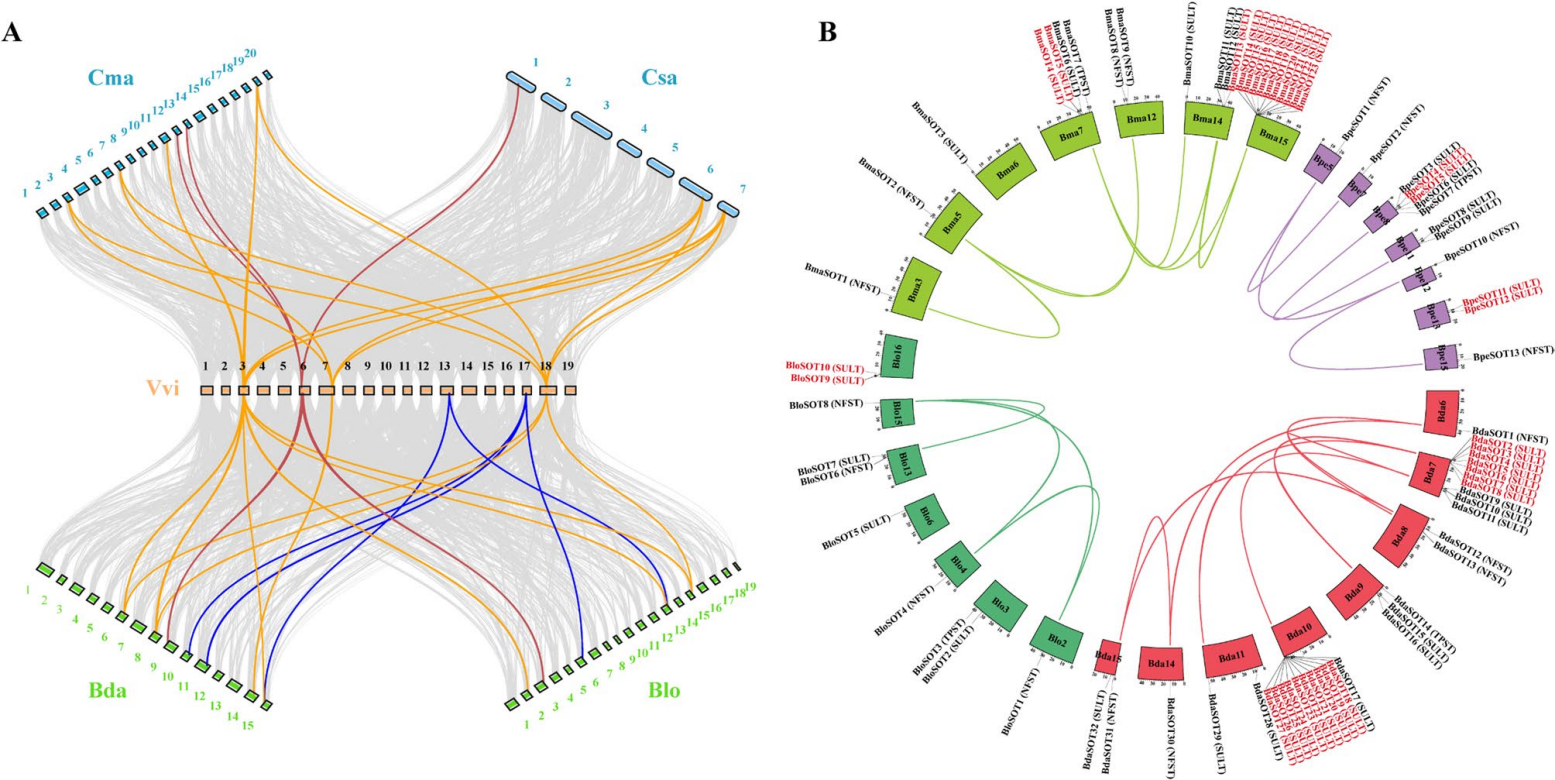
Synteny analysis of SOT genes among and within the selected genomes.**A** Interspecific synteny relationship of SOT genes between grape (*V. vinifera*) and representative species from Cucurbitaceae (*C. sativus* and *C. maxima*) and Begoniaceae (*B. loranthoides* and *B. darthvaderiana*). Yellow, red and blue lines connected the homologous genes in the SULT, TPST and NFST subfamilies, respectively. **B** Intraspecific synteny relationship of SOT genes within the species in Begoniaceae. The color lines connected the paralogy gene pairs within the species in Begoniaceae. The gene highlighted in red letters represented tandem-arrayed SULTs

After diverging from grape (*V. vinifera*), cucumber (*C. sativus*) and pumpkin (*C. maxima*) experienced one or two additional polyploidization events, respectively, leading to a theoretical orthology ratio of 1:2 or 1:4 between *V. vinifera* and *C. sativus* or *C. maxima*. Pairwise synteny analysis revealed that each SOT in *V. vinifera* corresponded one or two SOTs in *C. sativus*, and corresponded two or three SOTs in *C. maxima*, indicating gene loss of SOTs occurred in *C. sativus* and *C. maxima* following the whole-genome duplication events. A similar trend was observed in Begoniaceae. Each SOT in *V. vinifera* corresponded to fewer SOTs in *B. loranthoides* and *B. darthvaderiana* than the theoretical orthology ratios, indicating gene loss of SOTs also occurred in these two species following the whole-genome duplication events. Notably, no syntenic genes or orthologous genes of grape SULTs were detected in *C. sativus* or *C. maxima*, respectively. In contrast, syntenic genes or orthologous genes of grape SOTs from all three subfamilies, including SULTs, TPSTs and NFSTs, were identified in *B. loranthoides* and *B. darthvaderiana*. The results indicated that SULTs were lost in Cucurbitaceae, such as in *C. sativus* and *C. maxima* (Fig. 7A, Additional file 1: Tables S23-24).

Intraspecific synteny analysis of SOTs was performed within Cucurbitaceae and Begoniaceae species, respectively. Due to the absence of SULTs in Cucurbitaceae, only paralogous TPSTs and NFSTs were detected within these species. No paralogous gene pairs were found in *B. hispida* (Additional file 2: Fig. S5, Additional file 1: Tables S25-26). Interestingly, despite the high abundance of SULTs in *B. loranthoides* and *B. darthvaderiana*, only a small proportion were syntenic to grape SULTs, indicating that most SULTs in these species are not orthologous to grape SULTs. To investigate the driving factors behind the expansion of SULTs in Begoniaceae and their contraction in Cucurbitaceae, paralogous SOT gene pairs were examined within species. Only a few paralogous SULTs pairs were found within Begoniaceae species (Fig. 7B, Additional file 1: Tables S27-28). Then, five types of gene duplication events were checked, including singleton duplication (SD), dispersed duplication (DD), proximal duplication (PD), tandem duplication (TD), and whole-genome duplication (WGD). In Begoniaceae, WGD along with TD contributed the abundance of SULTs. WGD contributed 37.50%, 30.43%, 53.85%, and 50.00% SULTs in *B. darthvaderiana*, *B. masoniana*, *B. peltatifolia* and *B. loranthoides*, respectively, whereas TD accounted for 53.13%, 56.52%, 30.77% and 20.00% SULTs in these four species (Additional file 1: Table S29). Consistently, tandem-arrayed SULTs were observed in all four Begoniaceae species. For example, 7 and 10 SULTs in *B. darthvaderiana* were arrayed on chromosome 7 and 10, respectively, while 2 and 11 SULTs in *B. masoniana* were clustered on chromosome 7 and 15, respectively. The results confirmed that, as a major driving factor of SULTs expansion, TD significantly contributed to the abundance and lineage-specific duplication of SULTs in Begoniaceae (Fig. 7B). In contrast, due to the absence of SULTs in Cucurbitaceae, the duplication types of TPSTs and NFSTs revealed that WGD was the main driving factor for the expansion of SOT gene family in Cucurbitaceae. Specifically, 100.00% of duplicated SOT gene pairs were generated by WGD in the *C. argyrosperma*, *C. maxima*, *C. moschata*, and *C. pepo* four species, implying a critical role of WGD in Cucurbitaceae (Additional file 1: Tables S29-30).

**Fig. 8:**
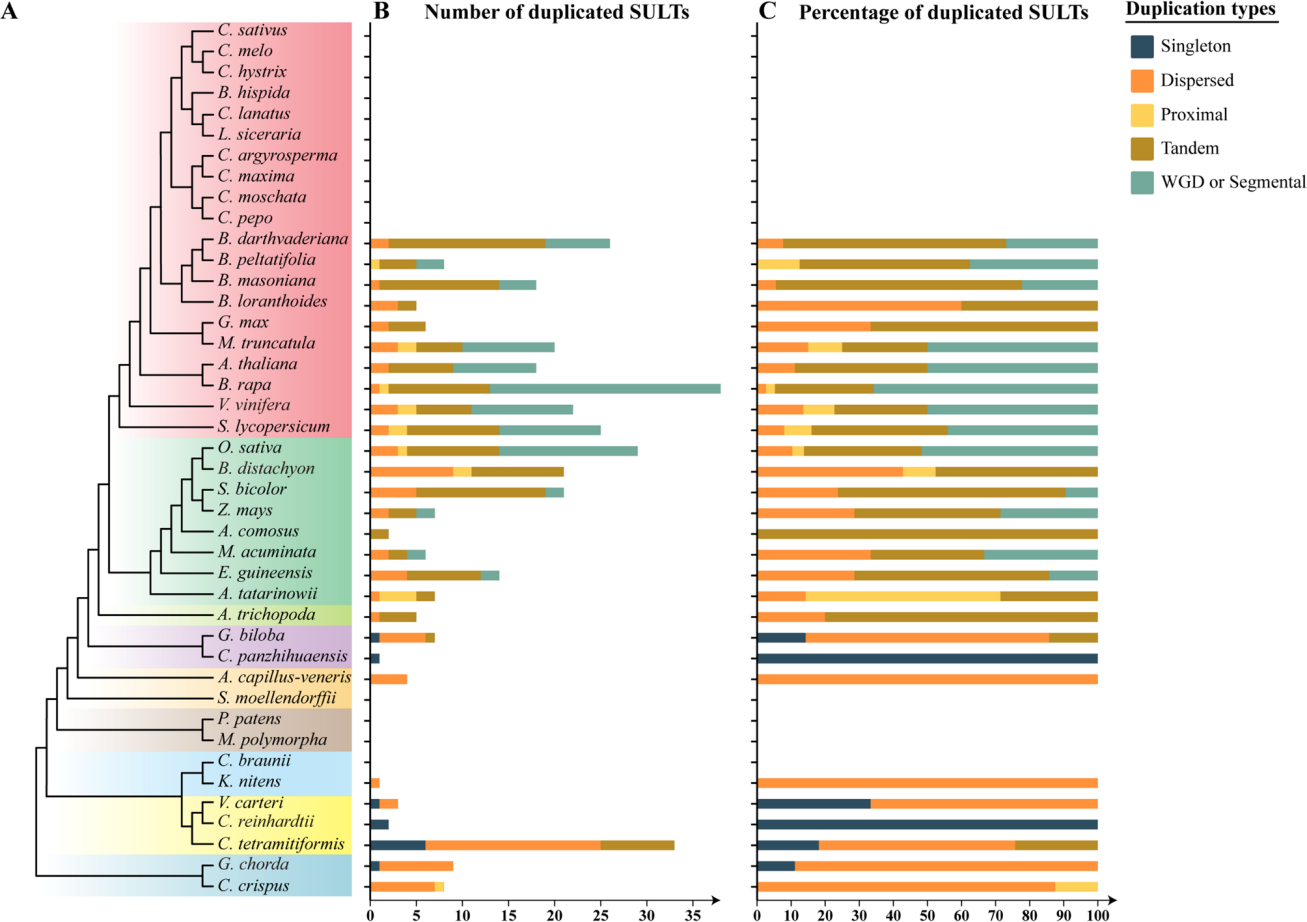
The number and percentage of duplicated SULTs derived from different types of gene.**A** Taxonomy tree of 42 plant species. **B** Number of duplicated SULTs. Five types of duplicated SULTs were identified, and the different colored bars represented the different types of SULTs duplication, including singleton duplication, dispersed duplication, proximal duplication, tandem duplication, and WGD or segmental duplication. **C** Percentage of duplicated SULTs produced by the five different duplication types

The synonymous substitution rates (Ks) of adjacent tandem-duplicated SULTs in Begoniaceae were calculated. The Ks values (less than 0.05) of TD-generated SULT pairs were much lower than those of WGD-generated pairs in Begoniaceae, further supporting a more recent origin of SULTs in Begoniaceae (Additional file 1: Table S31). Furthermore, analysis of duplication types in SULTs across 42 selected species revealed that TD played an important role in the expansion of SULTs in monocots and core eudicots (Fig. 8, Additional file 1: Tables S29-30).

### The impact of SULTs deficiency on sulfated products

Given that SULTs have been reported to sulfate low-molecular-weight metabolites, we investigated the potential impacts of SULT deficiency on sulfated metabolites in certain species using comparative genomics and metabolic approaches. Metabolite profiling analysis was performed in the species studied in the current study by querying the PCMD database, which is built on a genome-scale metabolic model (GEM) and served as an effective tool for predicting the presence of metabolites. Since sulfated brassinosteroids (BRs), 12-sulfooxyjasmonate/12-hydroxyjasmonate sulfate, and quercetin 3-sulfate (a kind of sulfated flavonoids) have been reported as typical sulfated substances in plants, the distribution of the above sulfated substances was examined across representative land plant species. Meanwhile, sequenced plant genomes were analyzed to retrieve SOT sequences and sort out the SULTs by phylogenetic and domain analysis.

**Fig. 9:**
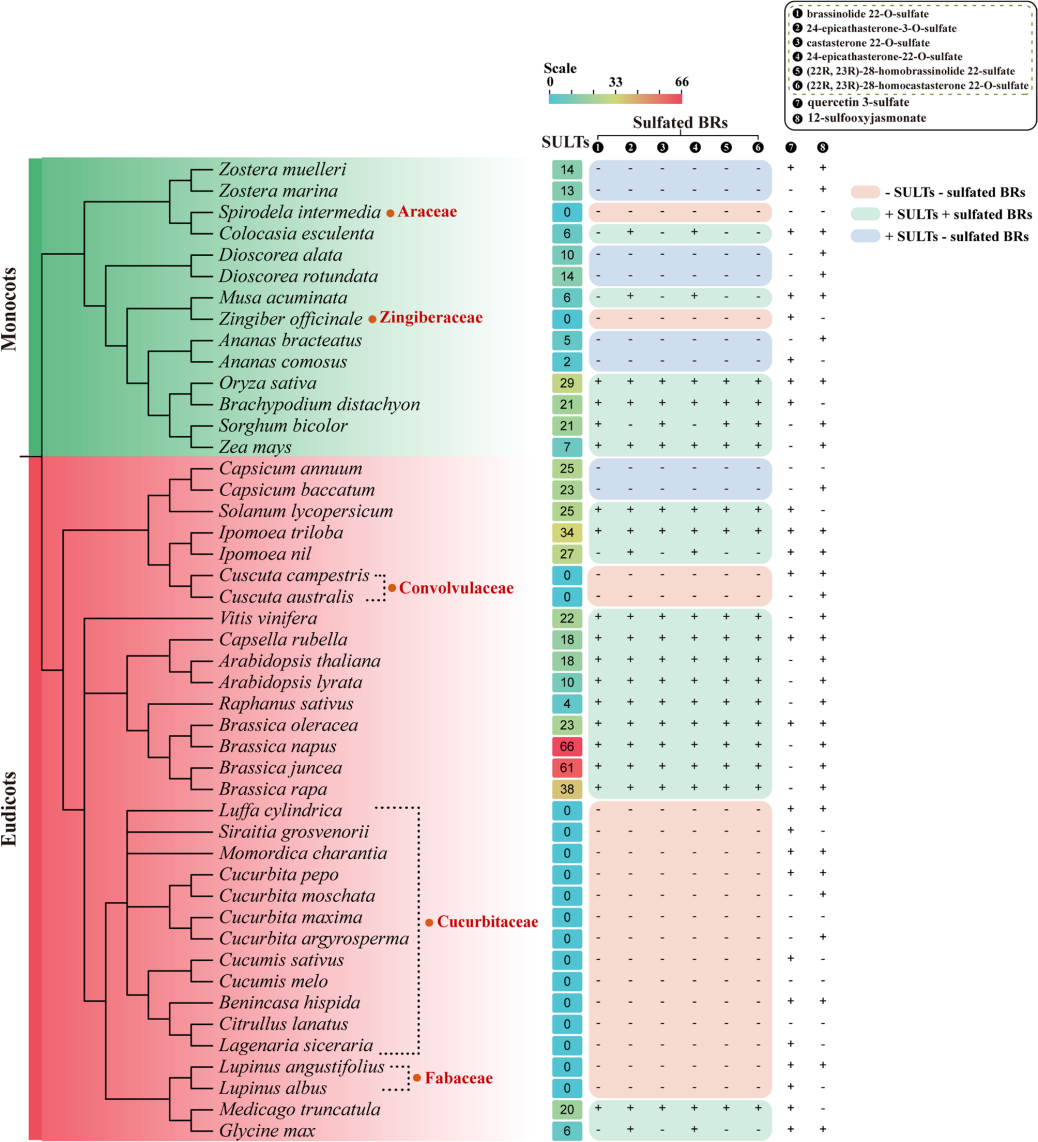
The distribution of SULTs and sulfated metabolites in the representative species of monocots and core eudicots Metabolite profiling was performed among the representative species of monocots and core eudicots by querying the PCMD database, and the presence of sulfated BRs, 12-sulfooxyjasmonate, and quercetin 3-sulfate were checked based on comparative metabolomics under a genome-scale metabolic model (GEM). Meanwhile, the SULTs within each species were identified and quantified by phylogenetic and domain analysis. Based on the relationship between SULTs and sulfated BRs, these species were classified into three categories and displayed with three background colors, respectively. - SULTs - sulfated BRs: absence of SULTs and sulfated BRs; + SULTs + sulfated BRs: presence of both SULTs and sulfated BRs; + SULTs - sulfated BRs: presence of SULTs but absence of sulfated BRs

Taking sulfated BRs as an example, these species were classified into three categories based on the relationship between SULTs and sulfated BRs: (1) absence of SULTs and sulfated BRs (- SULTs - sulfated BRs); (2) presence of SULTs and sulfated BRs (+ SULTs + sulfated BRs); and (3) presence of SULTs but absence of sulfated BRs (+ SULTs - sulfated BRs). The first category (- SULTs - sulfated BRs) encompassed all 12 Cucurbitaceae species studied here, along with certain species from other genera, such as *Lupinus albus*, *Lupinus angustifolius*, *Cuscuta australis*, *Cuscuta campestris*, *Zingiber officinale*, and *Spirodela intermedia*, indicating this pattern is likely widespread across the entire Cucurbitaceae family. In contrast, a variety of unmodified BRs and conjugated BRs with other modifications, such as glycosylated and hydroxylated BRs, were distributed in Cucurbitaceae species. The absence of sulfated BRs, coupled with the presence of unmodified, glycosylated, and hydroxylated BRs, implied that the lack of sulfated BRs is largely due to the absence of corresponding SULTs in Cucurbitaceae. The second category (+ SULTs + sulfated BRs) was observed in many species of monocots and core eudicots, particularly in Brassicaceae. This may be attributed to the abundance of sulfated compounds, such as glucosinolates in Brassicaceae plants. The third category (+ SULTs - sulfated BRs) was observed in specific species, including *Ananas comosus*, *Ananas bracteatus*, *Zostera marina*, *Zostera muelleri*, *Dioscorea alata*, *Dioscorea rotundata*, *Capsicum baccatum*, and *Capsicum annuum*. However, this trait was limited to certain species or genera and not shared across their entire families. Further studies, including enzymic, functional, metabolomic, and genetic analyses, are needed to determine whether the expansion of SULTs is associated with the sulfation of other unknown metabolites (Fig. 9, Additional file 1: Table S32).

Moreover, 12-sulfooxyjasmonate and quercetin 3-sulfate were distributed in four out of ten Cucurbitaceae species studied in this study, respectively, although SULTs were entirely absent in these species. 12-sulfooxyjasmonate was distributed in *C. argyrosperma*, *C. moschata*, *C. pepo*, and *B. hispid*, while quercetin 3-sulfate was distributed in *C. sativus*, *C. pepo*, *B. hispida* and *L. siceraria*. This raised the question of which SOTs were responsible for sulfating these metabolites in Cucurbitaceae. Taking cucumber CsaSOTs protein as examples, domain analysis by querying the InterPro database provided insights. Although both CsaSOT1 and CsaSOT2 belong to the NFST subfamily (IPR052796), CsaSOT2, but not CsaSOT1, was predicted to contain an additional Sulfotransfer_1 domain (IPR000863, PF00685), implying that CsaSOT2 may have broader substrate specificity, and NFSTs with Sulfotransfer_1 domain may play important roles in sulfonating small molecules. Similar findings were obtained across all ten Cucurbitaceae species, with a portion of NFSTs in each species predicted to possess an additional Sulfotransfer_1 domain (IPR000863, PF00685) (Additional file 2: Fig. S6). The domain architecture of Cucurbitaceae NFSTs was detailed in Additional file 1: Table S22. Currently, LC-MS/MS-based metabolomic and enzymatic analyses are hindered due to the unavailability of key sulfated reference standards, such as sulfated BRs and 12-sulfooxyjasmonate, which remain labeled as “not experimentally verified” in the PCMD database. Further genetic, functional and enzymatic evidence are required to clarify the exact function and substrates of NFSTs in plants.

## Discussion

### The ancient origin, classification, and nomenclature of the SOT gene family in plants

Sulfotransferases (SOTs) have been reported to be widespread in a variety of life forms, spanning prokaryotes and eukaryotes. These enzymes catalyze the transfer of a sulfuryl group from the PAPS donor to a hydroxyl group of various substrates. Sulfated products become more water soluble than non-sulfated molecules, thus sulfation usually facilitates bioactivation and excretion on their substrates. Currently, SOTs in metazoans are clearly classified into three main groups for sulfating different kinds of substrates, including SULTs for metabolites, such as endocrine hormones, drugs, neurotransmitters, and polyphenols, TPSTs for proteins, as well as CHSTs for glycosaminoglycans [30]. In plants, there is no systematic nomenclature for plant SOTs. Current identifications, classifications and nomenclature of SOTs are primarily limited to the SULT group, even in the model plant *Arabidopsis thaliana*. Although some researchers have classified plant SOTs into subfamilies according to the nomenclature system in animals, some well-characterized plant SOTs with identical substrate specificities were not consistently clustered into the same subfamilies. Plant SOTs were also investigated for their involvement in retrograde signaling by producing PAP as by-products, and divided into two subgroups, SOTs (SOT1-18) and TPSTs/SOTs (TPST and its related SOT19-21) [31]. However, AthSOT19-21 were annotated as “nodulation-related proteins” in NCBI, with no reported substrate information. AthTPST was added to the SOT gene family due to its ability to sulfate small peptides such as PSK and PSY1 [23, 32]. Given the complexity of SOT gene family members, grouping these diverse enzymes into subfamilies remains a significant challenge.

In this study, a combination of HMM and BLASTP searching methods were used to identify sulfotransferases in plants, and a total of 536 SOT genes were retrieved from 42 representative plant lineages. Furthermore, a large-scale analysis of sulfotransferases occurrence was performed by querying the InterPro database with corresponding IPR numbers, and a comprehensive dataset of SOTs sequences was created. Multiple proteins with representative domains under different IPR numbers were associated to SOTs based on function annotations in the InterPro database. Phylogenetic, conserved domain, subcellular localization and synteny analyses in the current study revealed that SOTs were initially divided into two groups, membrane-bound proteins and soluble cytosolic proteins. Membrane-bound SOTs were further divided into two groups based on substrates specificity, one group targeting tyrosylproteins with a nomenclature of TPSTs, and the other group targeting nodulation factors or similar substrates with a nomenclature of NFSTs. Soluble cytosolic sulfotransferases, termed SULTs, target low-molecular-weight metabolites. CHSTs were widespread in metazoans and bacteria, whereas were entirely absent in land plants. Therefore, we divided plant SOTs into three subfamilies, including TPSTs, NFSTs and SULTs.

We attempted to further classify subgroups within each subfamily. Given the limited gene number and small subfamily size of TPSTs and NFSTs, they were not subdivided. SULTs, highly abundant in most plant species, are a main factor influencing the size of the SOT gene family among different species. However, it is very difficult to divide SULTs into subgroups. In our study, some well-characterized plant SOTs with identical substrate specificity were not clustered into the same subgroups and SULTs from the same species formed distinct clusters in the phylogenetic tree (Additional file 2: Fig. S2 and S3), which is consistent with that in previous studies [26]. For example, 17 AthSOTs did not cluster with any *O. sativa* SOT genes in phylogenetic analyses [25]. These findings indicated that the SULT subfamily may have experienced lineage-specific evolution during the diversification of monocots and core eudicots. In plants, both sequence similarity and substrate specificity could influence the formation of subgroups, making it difficult to establish an optimal criterion for subgroup classification within the SULT family.

Although our study has advanced the classification of plant SOTs, the diverse nature of the family continued to pose challenges for a totally definitive and simplified categorization. Current classification may not fully capture the complex evolutionary and functional relationships within this family. Clarification substrate specificity through exact enzymatic mechanisms and additional genetic studies may enable a deeper understanding and more accurate classification of this gene family in the future.

### Horizontal gene transfer and evolution of NFSTs in plants

Horizontal gene transfer (HGT), the movement of genetic material between organisms by asexual means, is a major driver of genome evolution in bacteria and archaea. Recent studies have shown that HGT between bacteria and eukaryotes is also not rare and played crucial roles in shaping the evolution of land plants. Bacteria have been demonstrated to be the most important sources of new genetic sequences for eukaryotes through HGT. Genes acquired via HGT could compensate for the functional loss of other genes or provide new functionality, thereby enhancing the fitness and adaptability of the recipient organism [33–35]. However, HGT is known to be a serious disturbing factor to estimate a correct phylogeny, and constructing species-phylogenetic trees has become an important method for identifying mislocated HGT genes based on taxonomic relationship.

In this study, phylogenetic analysis combined with high-throughput screening suggested plant NFSTs might be acquired from bacteria through an HGT event. Nod factors, produced by nitrogen-fixing bacteria, play important roles in the symbiotic relationships between certain plants and nitrogen-fixing bacteria. Their basic structure consists of a chitooligosaccharide (a chain of β−1,4-linked N-acetylglucosamines) linked to an acyl chain. NodH sulfotransferase facilitates the sulfation of nod factors by transferring sulfate from PAPS to the terminal 6-O position of Nod factors [36]. By using AthSOTs amino acid sequences as bait, we identified multiple nodulation factor sulfotransferases (NFSTs, IPR052796) through BLASTP searches of relevant genomes. Furthermore, a large-scale analysis of the occurrence of NFSTs through querying the InterPro database using IPR052796, revealed that NFSTs are ubiquitously distributed in plants and bacteria, but absent in animals and fungi. This finding raised the hypothesis that the first plant NFST originated from an HGT event from bacteria during the origin of plant life forms. Phylogenetic analysis showed that plant NFSTs and bacteria NFSTs formed a distinct clade with high bootstrap support. Within this clade, green algal NFSTs closely clustered with bacterial NFSTs. The results suggested that the HGT of NFSTs from bacteria likely occurred prior to plants invaded the land, although most HGT events associated with plants took place during the early phase of streptophytes evolution or during the origin of land plants.

Gene structure analysis in this study revealed that bacterial and green algal NFSTs uniquely share intron-less architectures, providing genomic evidence supporting their structural conservation across these taxa. All NFSTs in other selected species contain introns, indicating that intron gain events may occur for NFSTs as adaptive responses to HGT during plant diversification (Fig. 3B). Most transferred DNA would become nonfunctional, but multiple strategies enable it to gain functionality in different instances. The acquisition of introns has been demonstrated to be an effective strategy for gaining functionality of transferred genes, by promoting the increased expression of transferred genes. Indeed, introns are commonly found in functional HGT genes in many eukaryotes [33, 35].

Additionally, we found that the distribution patterns of CHSTs and NFSTs showed significantly different trends. CHSTs and NFSTs were entirely absent in land plants and animals, respectively, although they were both identified in algae and bacteria. CHSTs are known to sulfate carbohydrates in animals. The substrate of NFSTs in plants remains unknown, even in the model plant *A. thaliana*. Establishing their specificity, functions and regulation requires further study. Bacterial NodH is the only NFST member with a known substrate, which is the nod factor with the acetylglucosamines as backbone. Since both CHSTs and NFSTs target substrates with glucose backbones, we wonder if the transferred NFSTs replaced CHSTs in plants, and gaining NFSTs from HGT enabled the loss of CHSTs function in plants. Genes obtained via HGT were reported to fall into two categories, retaining pre-existing functions or conferring new functionality, including enhanced the recipient nutrition, protection, and adaptation to extreme conditions [33, 34]. For example, the Nodulin 26-like intrinsic protein (NIP) family, acquired from bacteria through an HGT event, and evolved from bacterial arsenic efflux channels into essential nutrient transporters in seed plants by sub-functionalization. During this process, the nutritional demands of land plants acted as a key driver for the functional divergence of NIPs [37]. Bacterial NFSTs possess narrower substrate specificity than CHSTs. SULTs are absent in certain plant species, such as Cucurbitaceae species. There may be some plant-, lineage- or species-specific substates, not found in animals and bacteria, would be sulfated to render plants better environmental adaptability. These above factors could drive the neo- or sub-functionalization of NFSTs, particularly in evolving broader substrate specificity to meet the sulfation demands of specific metabolites in certain plant species. It remains to be further confirmed that whether the transferred NFSTs have been employed by plants and underwent neo- and sub-functionalization for a broader substrate specificity, instead of just replacing or maintaining the functional loss of CHSTs in plants. Although it has been reported that small differences in the sequence led to wide variations in substrate specificity of SOTs, the in vivo function of sulfation or the sulfated compound, and catalytic mechanisms of plant SOTs remained challenging fields, which could also give new insights of the evolution of plant SOTs. In addition, identification of new sulfated metabolites and the mechanism for the SOT specificities will bring more clarity to the importance of plant sulfation pathways. The absence of CHSTs in land plants and NFSTs in animals, despite their presence in algae and bacteria, suggested alternative sulfation enzymes or potential functional compensation. Critical unanswered questions concern whether plant NFSTs evolved broader substrate specificity to compensate for CHSTs loss, and how sequence variations drive functional diversification. Addressing these questions will require comparative structural studies, comprehensive metabolomic profiling across species, and functional validation of NFSTs activities in plants.

Phylogenetic analyses in the current study indicated that plant NFSTs originated from ancestral bacteria genes, however the exact bacteria donor needs to be further determined, because multiple bacteria species from different genera clustered together in the tree. Hence, identifying the donor bacteria through additional genetic and functional investigations remains an interesting task for us.

### Lineage-specific expansion of SULTs in plants

The most important HMM referred to SULTs is the SOT domain Sulfotransfer_1 (IPR000683, PF00685), with a ubiquitous occurrence in viridiplantae, metazoa and bacteria, and a greatly difference on the family size among species. A major finding of this study was that the absence of SULTs resulted in a reduced total number of SOTs within 10 Cucurbitaceae species. In contrast, the abundance of SULTs contributed the expansion of SOTs in the close Begoniaceae within the same order Cucurbitales. Gene gain and loss analysis showed that no gene duplications were detected during the recent species differentiation and formation of the 10 Cucurbitaceae species, but gene loss events were notably frequent.

Gene duplication events, particularly whole genome duplication (WGD) and tandem duplication (TD), are major evolutionary forces shaping biosynthetic genes responsible for the production and diversity of plant-specific metabolites [38–40]. For example, TD contributed the functional divergence of N-methyltransferase in caffeine biosynthesis, and BBEL genes in the corydalis-specific biosynthesis of cavidine [41, 42]. In this study, we demonstrated that TD greatly contributed the evolution of the SULT subfamily of plant SOTs. Both intraspecific and interspecies synteny of SOTs were detected only within each subfamily and no syntenic gene pairs were found across subfamilies, indicating different subfamilies underwent independent evolutionary trajectories. Although SULTs were highly abundant in Begoniaceae, they were not orthologous of grape SULTs and not products of WGD. Instead, TD was the main factor driving SULTs expansion in Begoniaceae. The Ks values (usually less than 0.05) of adjacent tandem-duplicated SULTs are much lower than those produced by WGD in Begoniaceae, further supporting a more recent origin of SULTs in Begoniaceae (Additional file 1: Table S31).

We further investigated the occurrence and expansion of SULTs on a large-scale. Paralogous SULT gene pairs were identified within 30 representative species, and their duplicated types were examined. Similar findings confirmed that TD significantly contributed to the divergence of plant SULTs during recent evolution with small Ks values. The expansion and contraction of the SOT family were largely attributed to the expansion of the SULT subfamily. Consistently, phylogenetic trees showed SULTs from the same species tend to cluster together, indicating species-specific clustering and lineage-specific expansion of plant SULTs. This phenomenon has also been observed by other researchers, as SOTs from Arabidopsis did not cluster together with that from rice into the same subfamilies [25, 26].

TD has been reported to be essential for the maintenance of large gene families, which could rapidly expand or contract in response to changing demands [43]. Since SULTs have been shown to sulfate low-molecular-weight metabolites, exploring the relationship between the lineage-specific expansion or contraction of SULTs and the diversity of sulfated metabolites across species, particularly in monocots and core eudicots, will allow us to better understand the functional and evolutionary strategies of plant SOTs.

### Correlation between lineage-specific evolution of SULTs and the diversity of sulfated metabolites in plants

Plant SOTs research remains a challenging field in linking specific genes to their metabolic products. A significant gap in our understanding lied in the substrate specificity of SOTs, which was hindered by several limiting factors. First, overlapping substrate spectra and different substrates specificity were observed among different SULT in in-vitro biochemical assays. For example, on the one hand, flavonoids have been reported to be sulfated by AtSOT5, AtSOT8, AtSOT12, and AtSOT13. On the other hand, AtSOT12 exhibited broad substrate specificity ranging from flavonoids to plant hormones, such as brassinosteroids and salicylic acid, whereas AtSOT15 had a much narrower narrow substrate specificity, primarily sulfating 12-hydroxyjasmonate [15, 17–19]. Second, current knowledge of substrate specificity was largely derived from in-vitro enzymatic assays through quantification of the transfer of a PAP^35^S sulfo group to various substrates by recombinant SULT proteins. However, in-vitro substrate specificity does not always reflect in-vivo activity. For example, overexpression of AtSOT10 in Arabidopsis did not result in any characteristic brassinosteroids-deficient phenotypes, and loss-of-function mutant of *atsot10* did not display enhanced hypocotyl growth, although AtSOT10 could sulfate brassinosteroids in-vitro [44]. Third, it should be noted that naturally occurring sulfated brassinosteroids or 12-sulfooxyjasmonate have not been detected in plants. The levels of endogenous sulfated metabolites are typically very low under normal conditions and are only induced under specific conditions. Additionally, the lack of sulfated compounds as the standard made it difficult to identify and quantify these metabolites in natural sources [11]. Forth, if the overlapping substrate spectra and different substrates specificity among different SULTs are indeed accurate, it becomes very difficult to dissect the function of individual SULTs and their respective substrates, determine in vivo substrate specificity, and establish the correlation between the function of a SULT and its sulfated metabolites.

Due to the complexity and limitations of current research on SOTs and their substrates, we employed comparative genomics and metabolic approaches to explore the relationship between SOTs and their substrates. The PCMD database was developed under a genome-scale metabolic model (GEM) and integrated genomic resources from 530 plant species, with compounds/enzymes information from MetaCyc, RefMetaPlant and KEGG databases. This database enables the prediction of metabolites and reactions of those 530 plant species [45]. We performed metabolite profiling analysis by querying the PCMD database, and found that different plants may have evolved distinct metabolite profiles to adapt to specific environmental or developmental conditions. Notably, the lack of SULTs along with limited sulfated metabolites was observed in certain species from diverse families and genera in monocots and core eudicots, particularly in all 12 Cucurbitaceae species studied in the current study. The results indicated that the absence of SULTs significantly impacted the production of corresponding metabolites. Intriguingly, PCMD database queries revealed that 12-sulfooxyjasmonate and quercetin 3-sulfate were distributed in a limited number of SULT-deficient Cucurbitaceae species. Domain analysis via the InterPro database showed that some NFSTs possessed an additional Sulfotransfer_1 domain (IPR000863, PF00685), providing the possibility for NFSTs with broader substrate specificity (Additional file 2: Fig. S2). However, whether the loss of SULTs enables NFSTs to acquire broader substrate specificity in SULT-deficient species, and whether the evolutionary changes in SULTs and NFSTs represent adaptations to specific sulfated products and specific environmental conditions in these species, remain open questions that require further investigation.

Additionally, the abundance of SULTs but lacking of sulfated brassinosteroids was observed in certain species, indicating a possibility of the presence of unknown sulfated metabolites. It has also been reported that finding novel sulfated metabolites is very possible. For instance, *Eucalyptus* possesses a great number of SOTs than Arabidopsis, yet no sulfated compounds have been known in this species to date. Identification of novel sulfated metabolites across a broader range of plant species in the future may provide deeper insights into the functional significance and biological roles of plant SOTs. Feeding mutant and wild-type plants with S^35^, followed by mass spectrometry analysis would be a more promising and informative approach for identifying novel sulfated metabolites. On the other hand, the discovery of novel sulfotransferases is also very possible, even in the well-studied model organism Arabidopsis. For instance, the knockout mutant of *tpst* exhibited only a mild phenotype, implying the potential existance of additional copies of this TPST protein [23].

Our findings revealed a complex relationship between SULTs gene presence and sulfated metabolite profiles. In Cucurbitaceae species (e.g., Cucumis melo and Citrullus lanatus), SULT genes were detected whereas certain sulfated metabolites (e.g., sulfated brassinosteroid) were absent in PCMD database queries, with the opposite pattern observed in other taxa. This discrepancy suggested that the relationship is more complex than a simple direct correlation and may involve the presence of unknown sulfated metabolites or alternative mechanisms for the functionality or absence of specific compounds. To address these complex relationships, we propose integrating advanced metabolomics with high-resolution mass spectrometry, CRISPR-based functional validation, and multi-omics correlation studies in the future. This strategy should clarify whether discrepancies originate from undetected compounds, alternative sulfation pathways, or condition-specific enzyme activities, ultimately providing a more comprehensive understanding of plant sulfation networks.

Additionally, whether the loss of SULTs enables NFSTs to acquire broader substrate specificity in SULT-deficient species, and whether the evolutionary changes in SULTs and NFSTs represent adaptations to specific sulfated products and specific environmental conditions in these species, remain open questions that require further investigation. The deficiency of sulfotransferases (SULTs) in Cucurbitaceae may modulate plant development and adaptive responses by altering the production and localization of sulfated compounds. Taking BRs as an example, NFSTs do not appear to fully compensate for SULTs function in Cucurbitaceae species lacking SULTs, as no sulfated BRs were detected in these species based on current query results from the PCMD database. SULTs are known to sulfate BRs to attenuate their activity. The absence of SULTs in Cucurbitaceae species may lead to two potential metabolic consequences. First, BRs modification may shift to alternative pathways such as glycosylation or acylation, resulting in enhanced alternative modifications of BRs. Alternatively, the loss of SULTs could result in elevated levels of free BRs in Cucurbitaceae species, thereby influencing their growth and development. For instance, whether the rapid coiling of cucumber tendrils and abnormal fruit morphogenesis and irregular cavity enlargement in fruits are promoted by heightened free BRs. To clarify these issues, an integrated approach combining in-vitro biochemical assays, in-vivo genetic studies, and advanced metabolomics techniques is indispensable. Such a multidisciplinary strategy will be essential for elucidating the precise functions of plant SOTs and their substrates, ultimately advancing our understanding of metabolites sulfation and its contribution to plant environmental adaptability.

## Materials and methods

### Sequence retrieval and identification of SOT genes

To obtain plant SOTs, a total of 42 plant genomes were searched using combined Hidden Markov Model (HMM) and BLASTP searching methods. Firstly, a dataset was created by HMMER 3.0 searing against those genomes under 9 PFAM numbers (PF00685, PF03567, PF13469, PF05935, PF14269, PF09037, PF06990, PF17784, PF19798). An E value of 1e-3 was used as the threshold. Secondly, another dataset was obtained using BLASTP searching. Sequences of SOTs from *A. thaliana* were downloaded from the TAIR database, then all the AthSOTs were used as queries for the BLASTP searching against the 42 plant genomes with an E value of 1e-3. Finally, a union of the above two datasets were collected, and putative SOT genes were subjected to the InterPro database to verify conserved domains. For multiple transcripts with 100% identity within each species, the longest transcript was selected as the representative sequence to reduce redundancy. The SOTs obtained in this study were listed in Additional file 1: Table S1.

### Phylogenetic analyses

The sequences with a length shorter than 200 amino acids were removed to ensure the quality of phylogenetic reconstructions. The full protein sequences of SOTs were aligned using MAFFT (v7.526) [46] with default parameters. Sequence trimming was carried out by TRIMAL with the parameter set to 0.5. The multiple sequence alignment file was then submitted to IQ-TREE (version 2.3.6) software for the construction of the phylogenetic tree using the maximum-likelihood method [47]. The bootstrap value was 1,000. Finally, the phylogenetic tree was visualized by iTOL software (v7) [48].

### Conserved motif and gene structure analysis

The MEME (https://memesuite.org/meme) online tool was used to analyze the conserved amino acid sequences of SOTs under default parameters, and the number of defined motifs was set to 10. Gene Structure View (Advanced) embedded in TBtools [49] was used to predicted the numbers of exons and introns, and the SOT gene structures.

### Prediction of horizontally transferred genes

HGTector v2.0b3 [50] was employed to detect putative HGT genes of bacterial origin in *C. tetramitiformis*. For this analysis, we constructed a customized database containing RefSeq protein sequences from two taxonomic groups, including bacteria (eubacteria, TaxID: 2) and Chlorophyta (green algae, TaxID: 3041), which were retrieved from NCBI and processed with DIAMOND. Whole-proteome DIAMOND searches were performed using *C. tetramitiformis* proteins as queries, with an e-value cutoff of 1 × 10⁻³. The clustering algorithm was configured to use the “bandwidth grid” option for bandwidth optimization, while all other parameters remained at the default settings. For HGT detection, *C. tetramitiformis* (TaxID: 36881) was defined as the self-group and Chlorophyta (TaxID: 3041) as the close-group to establish phylogenetic reference boundaries. Two thresholds for the normalized bit-scores were established through computational fitting: a close-group cutoff at 15.60 and a distal-group cutoff at 22.56. The normalized bit-score for each gene was calculated by summing the bit-scores of all hits within each group for a given protein and dividing by the bit-score of the query protein. Genes with bit-scores < 15.60 in close-group and > 22.56 in distal-group were assigned to the high-confidence category. Additionally, genes with distal-group scores < 22.56 and zero close-group alignment (bit-score = 0) were assigned to the medium-confidence category. All remaining genes were classified into the low-confidence category.

### Gene duplication and loss analysis

Based on the species-tree for this study and gene-tree of SOTs, Notung with default parameters was employed to analyze the duplication and loss of SOTs during evolution by reconciling the species tree and the gene tree from the selected 14 in Cucurbitales. Five gene duplication types were estimated for the paralogy SOT gene pairs using the embedded duplicate_gene_classifier sub-program in MCScanX [51], including singleton, dispersed, proximal, tandem, and whole genome/segmental duplication types. The results were submitted and visualized on the Chiplot website. Meanwhile, the adjacent SOT genes of the physical distance within 2 Mb in the same chromosome were also considered as tandem duplication.

### Syntenic analysis and Ks calculation

The genomic data of the selected species in the current study were aligned by BLASTP with an E-value of 1e-3, then the alignment results were submitted to MCScanX software for interspecific and intraspecific synteny analysis. Then, the syntenic SOT gene pairs were retrieved. Finally, the nonsynonymous and synonymous substitution rate (Ka and Ks) values of SOT gene pairs were calculated by the sub-program SimpleKa/KsCalculator embedded in WGDI [52] software.

### Subcellular localization of CsaSOT proteins

The CDS region of three CsaSOT was cloned and ligated into pHG vector using in-fusion technology. The resulting p35S: CsaSOT-GFP expression vector was transformed into the *Agrobacterium tumefaciens* strain GV3101, and finally expressed in the leaf epidermal cells of *Nicotiana benthamiana* through PEG-mediated transient transformation. The SYP31 (Syntaxin of plants31)-RFP expression vector was co-expressed as a *cis*-Golgi marker [23, 53]. For confocal microscope observation, images were collected under a Leica confocal microscope (Leica, Germany) with a 20× water lens and with argon laser excitation at 488 nm.

### Prediction of sulfated metabolites

The metabolite information of the selected species was obtained and downloaded from the PCMD website (https://yanglab.hzau.edu.cn/PCMD.1, accessed from February 15, 2025, to February 25, 2025), and self-written scripts were employed to extract corresponding sulfated metabolites. For sulfated brassinosteroids, a substance was identified as sulfated brassinosteroids if its name contained any of the following keywords, such as “brassinolide”, “teasterone”, “typhasterol”, or “cathasterone”, along with the keyword “sulfate”. The keywords of “12-sulfooxyjasmonate” and “quercetin 3-sulfate” were used to retrieve those two substances, respectively. The search terms were set up in case-insensitive. The search terms were set to be case-insensitive to ensure comprehensive retrieval of relevant data. Subsequently, the presence of each sulfated substance in these species was determined, and a heatmap was generated.

## Supplementary Information

Additional file 1: Table S1.Gene name and ID in the thirty selected species. Table S2.NFSTs under IPR052796 searched in the InterPro database. Table S3.NFSTs under IPR052796 from bacteria searched in the InterPro database. Table S4.Reviewed SULTs under IPR000863 searched in the InterPro database. Table S5.SULTs under IPR005331 searched in the InterPro database. Table S6.SULTs under PFAM13469 searched in the InterPro database. Table S7.SULTs under PFAM17784 searched in the InterPro database. Table S8.SULTs under PFAM19798 searched in the InterPro database. Table S9.SULTs under IPR053143 searched in the InterPro database. Table S10.SULTs under IPR010262 searched in the InterPro database. Table S11.TPSTs under IPR010635 searched in the InterPro database. Table S12.TPSTs under IPR037359 searched in the InterPro database. Table S13.TPSTs under IPR007734 searched in the InterPro database. Table S14.TPSTs under IPR026634 searched in the InterPro database. Table S15.CHSTs under IPR018011 searched in the InterPro database. Table S16.CHSTs under IPR052654 searched in the InterPro database. Table S17.CHSTs under IPR009729 searched in the InterPro database. Table S18.CHSTs under IPR051135 searched in the InterPro database. Table S19. Predicted candidate HGT genes in the high-confidence category. Table S20.Predicted candidate HGT genes in the medium-confidence category. Table S21. Alignment results of CteSOT48 and taxonomic information of donor proteins. Table S22.Gene name and ID in the sixteen selected species. Table S23.List of orthologous SOT gene pairs in Begoniaceae, Cucurbitaceae and *Vitis vinifera*. Table S24.Ka/Ks ratios of orthologous SOT gene pairs in Begoniaceae, Cucurbitaceae and *Vitis vinifera*. Table S25. List of paralogous SOT gene pairs in Cucurbitaceae. Table S26.Ka/Ks ratios of paralogous SOT gene pairs in Cucurbitaceae. Table S27.List of paralogous SOT gene pairs in Begoniaceae. Table S28.Ka/Ks ratios of paralogous SOT gene pairs in Begoniaceae. Table S29.Types of duplications of SULTs in the selected species. Table S30.List of duplicated SULTs with duplication type in the selected species. Table S31.Ks of adjacent tandem-duplicated SULTs in Begoniaceae. Table S32. The distribution of the corresponding metabolites in the representative species of monocots and core eudicots. Table S33.The information of genomes used in this study.

Additional file 2: Fig. S1 Rooted phylogenetic tree of plant and algal SOT genes. Fig. S2 Rooted phylogenetic tree of SOT genes in land plants. Fig. S3 Rooted phylogenetic tree of SULTs in land plants. Fig. S4 Predicted potential HGT candidate genes of bacterial origin in *Cymbomonas tetramitiformis*. Fig. S5 Synteny analysis of SOT genes in Cucurbitaceae. Fig. S6 Representative domain analysis of CsaSOT1 and CsaSOT2 in the InterPro database.

## Data Availability

No datasets were generated or analysed during the current study.
