# Supplementary material for: Identification and evolution of the plant sulfotransferase family
Source: BMC Genomics. 2025 Oct 8;26:895. doi: 10.1186/s12864-025-12117-4 (PMC12506393; doi:10.1186/s12864-025-12117-4)
Supplement: Supplementary file 1 — Additional file 1: Table S1.Gene name and ID in the thirty selected species. Table S2.NFSTs under IPR052796 searched in the InterPro database. Table S3.NFSTs under IPR052796 from bacteria searched in the InterPro database. Table S4.Reviewed SULTs under IPR000863 searched in the InterPro database. Table S5.SULTs under IPR005331 searched in the InterPro database. Table S6.SULTs under PFAM13469 searched in the InterPro database. Table S7.SULTs under PFAM17784 searched in the InterPro database. Table S8.SULTs under PFAM19798 searched in the InterPro database. Table S9.SULTs under IPR053143 searched in the InterPro database. Table S10.SULTs under IPR010262 searched in the InterPro database. Table S11.TPSTs under IPR010635 searched in the InterPro database. Table S12.TPSTs under IPR037359 searched in the InterPro database. Table S13.TPSTs under IPR007734 searched in the InterPro database. Table S14.TPSTs under IPR026634 searched in the InterPro database. Table S15.CHSTs under IPR018011 searched in the InterPro database. Table S16.CHSTs under IPR052654 searched in the InterPro database. Table S17.CHSTs under IPR009729 searched in the InterPro database. Table S18.CHSTs under IPR051135 searched in the InterPro database. Table S19. Predicted candidate HGT genes in the high-confidence category. Table S20.Predicted candidate HGT genes in the medium-confidence category. Table S21. Alignment results of CteSOT48 and taxonomic information of donor proteins. Table S22.Gene name and ID in the sixteen selected species. Table S23.List of orthologous SOT gene pairs in Begoniaceae, Cucurbitaceae and Vitis vinifera. Table S24.Ka/Ks ratios of orthologous SOT gene pairs in Begoniaceae, Cucurbitaceae and Vitis vinifera. Table S25. List of paralogous SOT gene pairs in Cucurbitaceae. Table S26.Ka/Ks ratios of paralogous SOT gene pairs in Cucurbitaceae. Table S27.List of paralogous SOT gene pairs in Begoniaceae. Table S28.Ka/Ks ratios of paralogous SOT gene pairs in Begoniaceae. Table S29.Types [file 12864_2025_12117_MOESM1_ESM.docx]

**
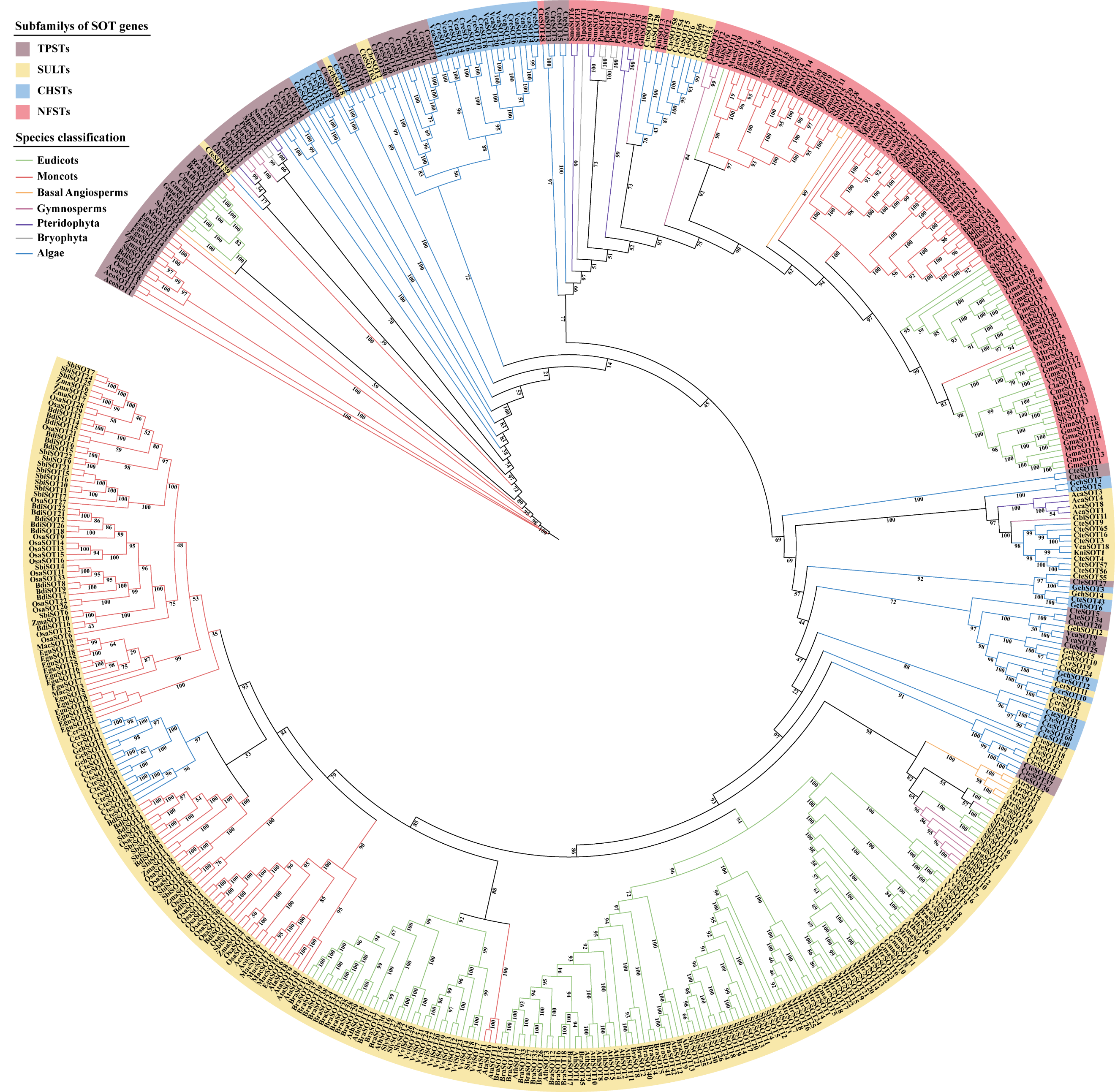
**

**Fig. S1 Rooted phylogenetic tree of plant and algal SOT genes.**

The tree was constructed using SOTs identiﬁed from 30 species in this study, including 2 species of red algae, 5 species of green algae, and 2 species of bryophyta, 2 species of pteridophyta, 2 species of gymnosperms, 1 species of basal angiosperms, 8 species of monocots, and 8 species of eudicots. The protein names in the tree were indicated by colored symbols corresponding to four subfamilies, including SULTs (yellow), TPSTs (brown), NFSTs (red) and CHSTs (blue). Colored branches were used to distinguish clades of eudicots, monocots, basal angiosperms, gymnosperms, pteridophyta, bryophyta, and algae. CHSTs were only found in algae and absent in land plants.


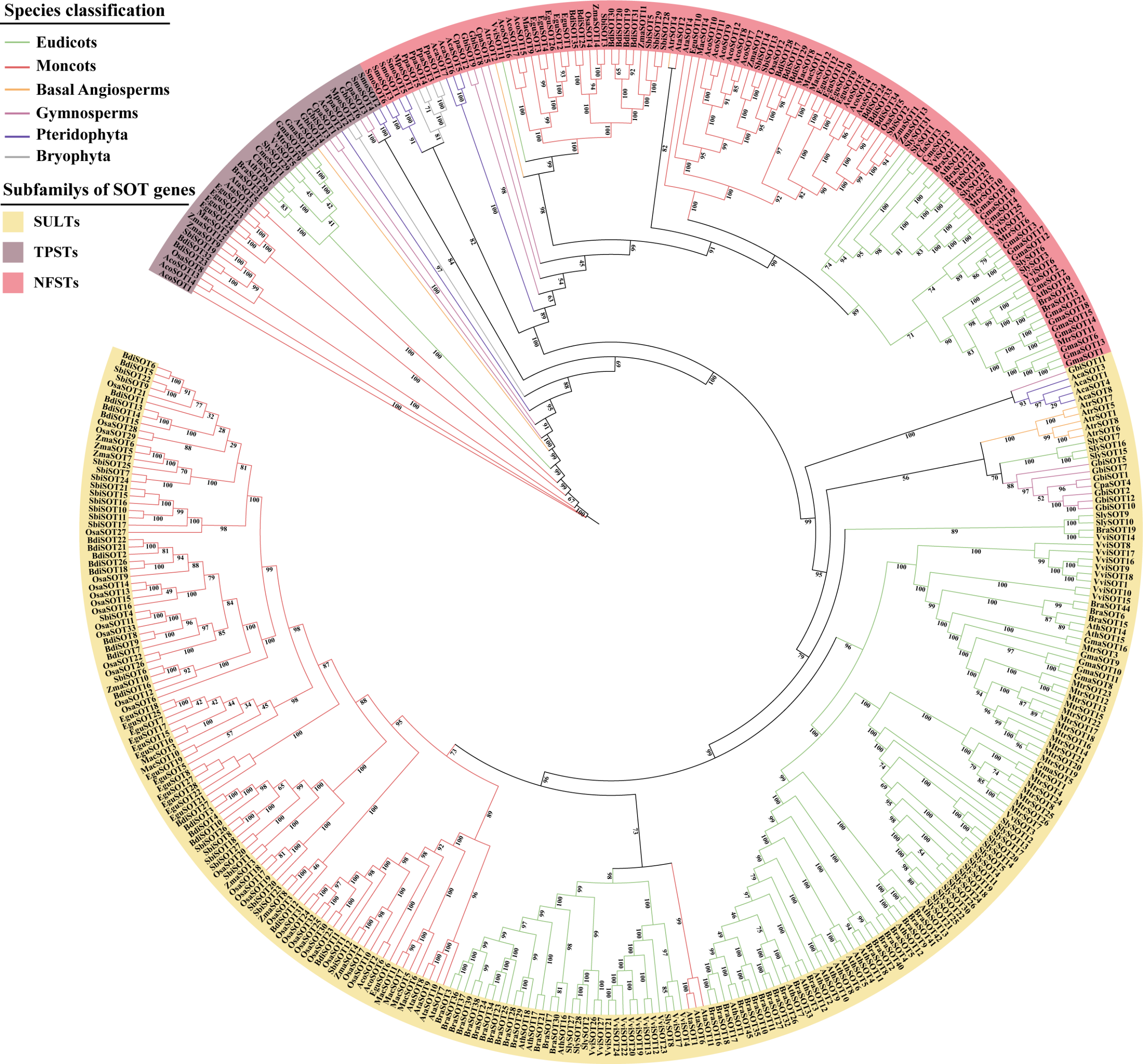


**Figure S2. Rooted phylogenetic tree of SOT genes in land plants.**

The tree was constructed using SOTs identiﬁed from land plants in this study, including 2 species of bryophyta, 2 species of pteridophyta, 2 species of gymnosperms, 1 species of basal angiosperms, 8 species of monocots, and 8 species of eudicots. The protein names in the tree were indicated by colored symbols corresponding to three subfamilies, including SULTs (yellow), TPSTs (brown), and NFSTs (red). Colored branches were used to distinguish clades of eudicots, monocots, basal angiosperms, gymnosperms, pteridophyta, and bryophyta.


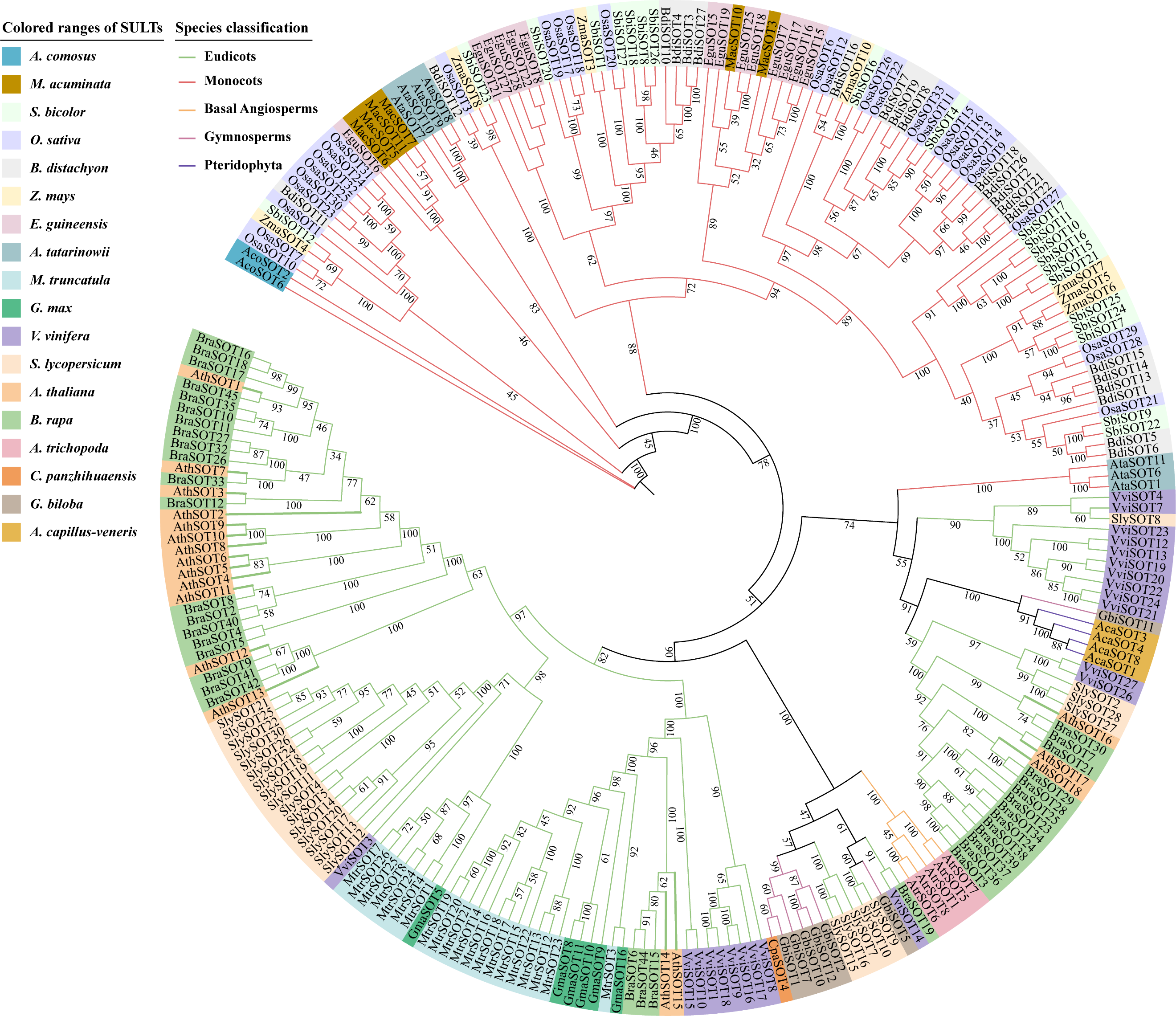


**Figure S3. Rooted phylogenetic tree of SULTs in land plants.**

The tree was constructed using SOTs identiﬁed from land plants in this study, including 2 species of bryophyta, 2 species of pteridophyta, 2 species of gymnosperms, 1 species of basal angiosperms, 8 species of monocots, and 8 species of eudicots. The protein names in the tree were indicated by colored symbols corresponding to three subfamilies, including SULTs (yellow), TPSTs (brown), and NFSTs (red). Colored branches were used to distinguish clades of eudicots, monocots, basal angiosperms, gymnosperms, pteridophyta, and bryophyta.

**
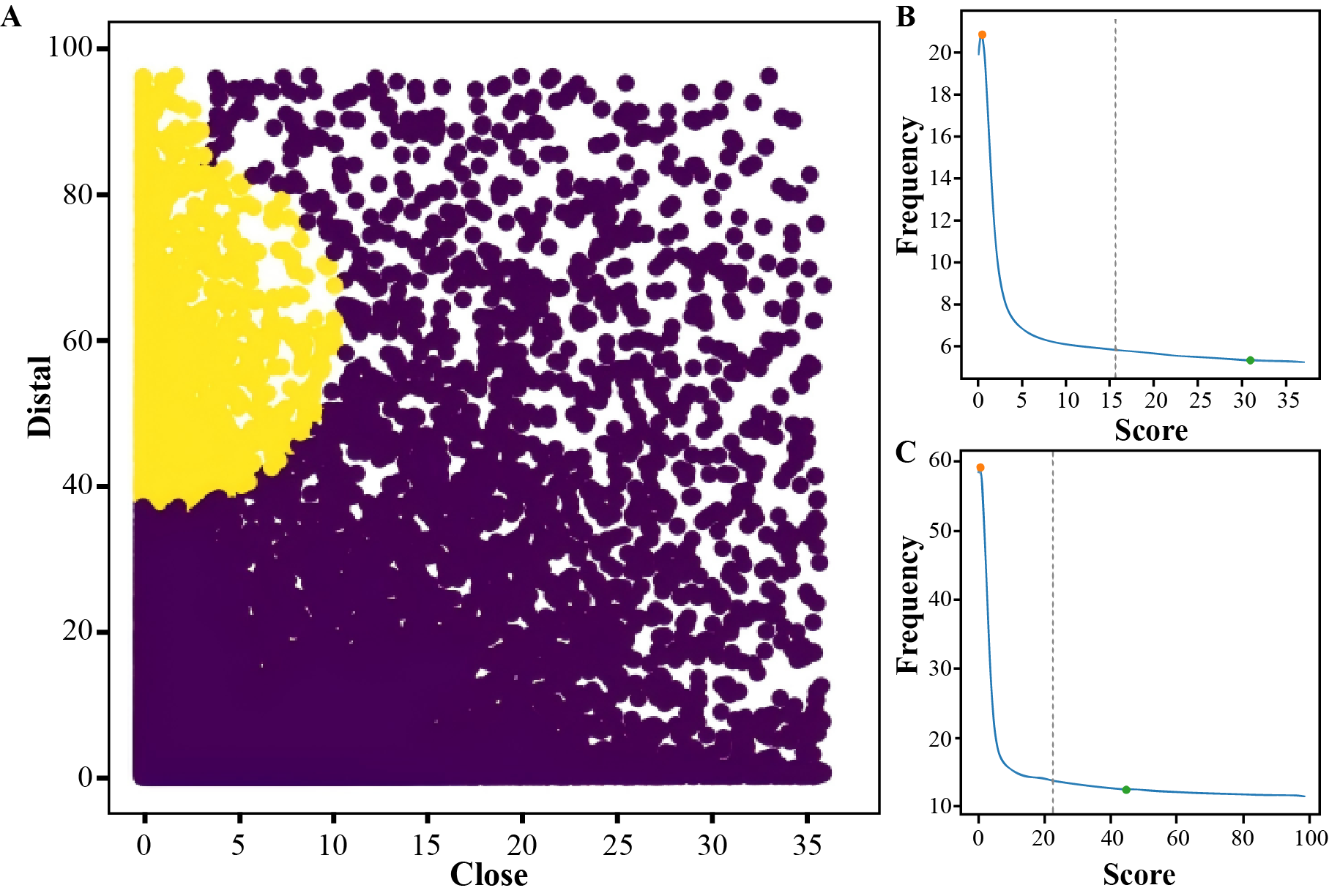
**

**Figure S4. Predicted potential HGT candidate genes** **of bacterial origin in** ***Cymbomonas tetramitiformis*.**

The HGT genes of bacterial origin in *Cymbomonas tetramitiformis* were predicted using the HGTector software.

**A)** A scatter plot showing the distribution of the genes in *Cymbomonas tetramitiformis* according to the normalized “close” and “distal” bit-scores. The normalized bit-scores were calculated by summing the bit scores of all hits within the close or distal group for each gene and dividing by the bit score of the query gene itself. These bit scores reflect the relative phylogenetic affinity of genes to close or distal taxonomic groups and assist in determining their potential horizontal transfer origin. Yellow dots: predicted HGT genes; Purple background: whole-genome distribution of *Cymbomonas tetramitiformis*.

**B)** Kernel density estimate (KDE) curve of the close group scores for all genes. The curve reflects their phylogenetic affinity within the specified close taxonomic group. **C)** KDE curve of the distal group scores. The curve reflects the divergence of genes from distal taxonomic groups.


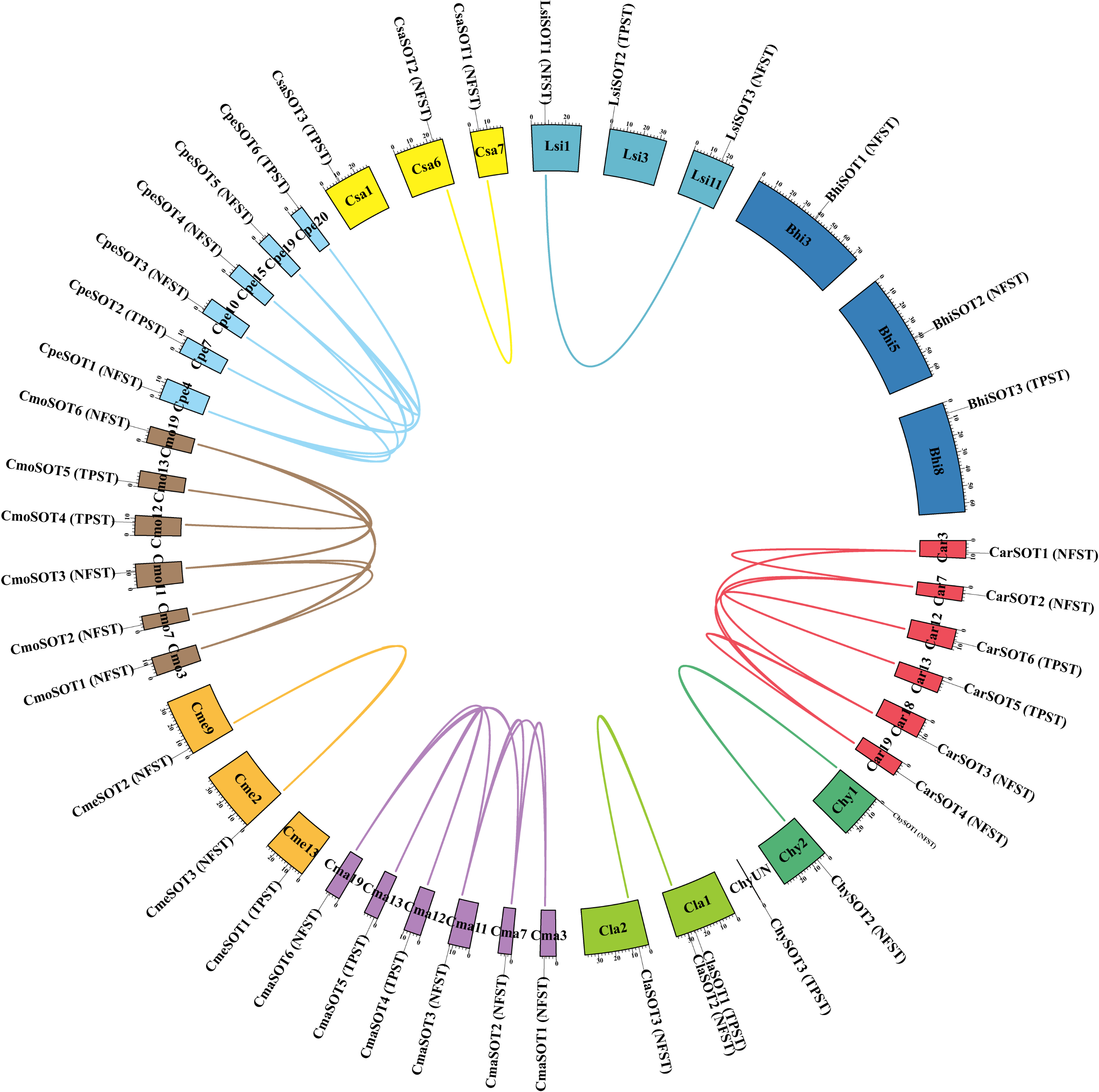


**Figure S5. Synteny analysis of SOT genes in Cucurbitaceae.**

Intraspecific synteny relationship of SOT genes within the species in Cucurbitaceae. The color lines connected the paralogy gene pairs within the species in Cucurbitaceae.

**
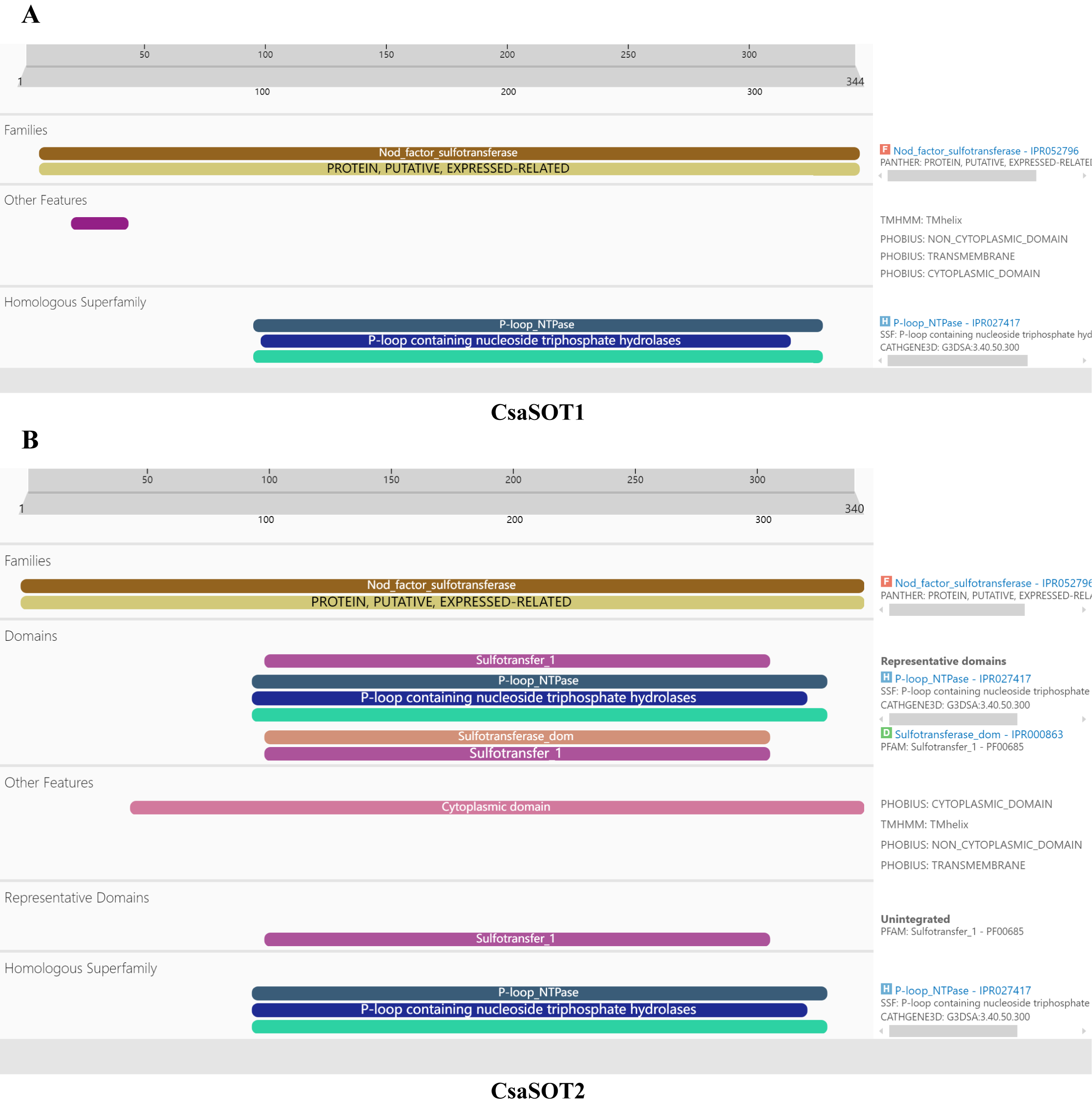
**

**Figure S6. Representative domain analysis of CsaSOT1 and CsaSOT2 in the InterPro database.**

**A)** Representative domain analysis of CsaSOT1. **B)** Representative domain analysis of CsaSOT2.
